# The RAVEN Toolbox and Its Use for Generating a Genome-scale Metabolic Model for *Penicillium chrysogenum*

**DOI:** 10.1371/journal.pcbi.1002980

**Published:** 2013-03-21

**Authors:** Rasmus Agren, Liming Liu, Saeed Shoaie, Wanwipa Vongsangnak, Intawat Nookaew, Jens Nielsen

**Affiliations:** 1 Department of Chemical and Biological Engineering, Chalmers University of Technology, Gothenburg, Sweden; 2 Center for Systems Biology, Soochow University, Suzhou, China; The Pennsylvania State University, United States of America

## Abstract

We present the RAVEN (Reconstruction, Analysis and Visualization of Metabolic Networks) Toolbox: a software suite that allows for semi-automated reconstruction of genome-scale models. It makes use of published models and/or the KEGG database, coupled with extensive gap-filling and quality control features. The software suite also contains methods for visualizing simulation results and omics data, as well as a range of methods for performing simulations and analyzing the results. The software is a useful tool for system-wide data analysis in a metabolic context and for streamlined reconstruction of metabolic networks based on protein homology. The RAVEN Toolbox workflow was applied in order to reconstruct a genome-scale metabolic model for the important microbial cell factory *Penicillium chrysogenum* Wisconsin54-1255. The model was validated in a bibliomic study of in total 440 references, and it comprises 1471 unique biochemical reactions and 1006 ORFs. It was then used to study the roles of ATP and NADPH in the biosynthesis of penicillin, and to identify potential metabolic engineering targets for maximization of penicillin production.

## Author Summary

Genome-scale models (GEMs) are large stoichiometric models of cell metabolism, where the goal is to incorporate every metabolic transformation that an organism can perform. Such models have been extensively used for the study of bacterial metabolism, in particular for metabolic engineering purposes. More recently, the use of GEMs for eukaryotic organisms has become increasingly widespread. Since these models typically involve thousands of metabolic reactions, the reconstruction and validation of them can be a very complex task. We have developed a software suite, RAVEN Toolbox, which aims at automating parts of the reconstruction process in order to allow for faster reconstruction of high-quality GEMs. The software is particularly well suited for reconstruction of models for eukaryotic organisms, due to how it deals with sub-cellular localization of reactions. We used the software for reconstructing a model of the filamentous fungi *Penicillium chrysogenum*, the organism used in penicillin production and an important microbial cell factory. The resulting model was validated through an extensive literature survey and by comparison with published fermentation data. The model was used for the identification of transcriptionally regulated metabolic bottlenecks in order to increase the yield in penicillin fermentations. In this paper we present the RAVEN Toolbox and the GEM for *P. chrysogenum*.

## Introduction

Genome sequencing projects have in recent years contributed enormously to our understanding of the metabolic capabilities of cellular systems. Functional annotation of the gene products allow for reconstruction of genome-scale metabolic models (GEMs) that summarize these metabolic capabilities in a consistent and compact way [1], [2]. A number of mathematical tools, including sampling of available metabolic states [3], [4] and methods borrowed from computational geometry [5], have been developed to analyze the resulting networks and to gain insight into the complex interactions that give rise to the metabolic capabilities. GEMs have also been used extensively for simulation of metabolism, particularly for metabolic engineering purposes [6], [7]. Since these models connect metabolites, proteins, and genes they are particularly well suited for the integration of metabolomics, proteomics, and genomics which is, in a sense, the goal of systems biology [8], [9].

The foundation of a GEM is the functional annotation of the genes. The first GEMs were primarily for model organisms for which direct evidence exists in the literature for a large proportion of the genomically encoded functions [10], . However, as the number of genome sequencing projects increases there is a growing demand of GEMs for less well known organisms. These models must by necessity be built largely relying on protein homology to more well-characterized organisms [12]. This, together with the large amount of manual work that is involved in a strict bottom-up reconstruction, has sparked interest in more automated approaches to model reconstruction. There are now a number of tools available for automated annotation of genes [13], [14]. However, the annotated genes must be linked to metabolic reactions in a way so as to generate a functional metabolic model. This includes the addition of spontaneous reactions and non-carrier mediated transport across membranes as well as sub-cellular localization of enzymes. Most importantly, the model must also be constructed in a way so that all reactions are balanced and well-connected [15]. This tends to become a problem if the gene-reaction relationship is automatically inferred from databases, partly due to differences in metabolite naming, but mainly because of how complex carbohydrates and complex lipids are represented. Because of the aforementioned issues it makes sense to use previously reconstructed models as templates for new GEMs. Here we present the RAVEN Toolbox, which allows the user to input GEM(s) for one or more template organisms, their corresponding protein sequences, and the protein sequences of the target organism. A GEM for the target organism is then constructed based on orthology between the protein sequences of the target organism and the organisms of the template models. Metabolic functions not present in the template models can obviously not appear in the new model, and to account for these missing reactions the RAVEN Toolbox also includes a functionality that matches proteins to KEGG Orthology (KO) categories [16] by using Hidden Markov models to capture the representative amino acid pattern in each KO. The resulting metabolic network can be used for automatic or manual gap filling, or it can be used on its own as a draft network.

Several approaches which also aim at generating GEMs from either a template model or from a general database have been published [17]–[20]. Table 1 summarizes the capabilities of the RAVEN Toolbox compared to some other published approaches when it comes to automatic reconstruction. However, the largest difference is maybe not in the approaches taken, but in that the RAVEN Toolbox is a complete software for all tasks involving reconstruction and simulation of GEMs. In this aspect the RAVEN Toolbox is more similar to the COBRA Toolbox, but with extensive reconstruction capabilities [21]. Even though the RAVEN Toolbox can be used for fully automated reconstruction, in a manner similar to Model SEED, the intended purpose is to make use of the extensive quality control and gap identification/gap filling features for increasing the quality of reconstructions, as well as for decreasing the time needed for reconstructing high-quality models.

**Table 1:** Comparison between the RAVEN Toolbox and other software for automatic GEM reconstruction.

	*RAVEN*	*Model SEED * [*20*]	*AUTOGRAPH * [*18*]	*IdentiCS * [*60*]	*GEM System * [*17*]
Includes general network	X	X		X	X
Generates functional models	X	X			
Assigns sub-cellular localization	X				
Can use user defined models	X		X		
Integrates gap filling	X	X			X
Offline software	X			X	
Includes visualization	X			X	X
Gene prediction				X	X

The RAVEN Toolbox was evaluated for its ability to reconstruct a GEM for the well studied yeast *Saccharomyces cerevisiae*, and the resulting GEM was compared with a manually reconstructed model. Thereafter the RAVEN toolbox was used for the reconstruction of a GEM of the industrially important mold *Penicillium chrysogenum*. The *Penicillium* genus encompasses species of great economical, medical, and environmental importance [22]. Members of the *Penicillium* genus serve important roles in the food industry, both as some of the main spoilers of fresh vegetables and as essential actors in the production of blue cheeses. Most importantly though, they are sources of major antibiotics, particularly penicillin and griseofulvin.

The industrial production of β-lactam antibiotics, such as penicillins and cephalosporins, is one of the success stories of biotechnology. Today the β-lactams represent one of the largest biotechnological products in terms of value, with sales of about USD 15 billion [23]. The industrial *P. chrysogenum* strains have been subjected to 50 years of directed evolution to increase the yields and titers of penicillin, with great cost reduction and productivity gain, but the yields are still far from the theoretical maximum [24]. A GEM of *P. chrysogenum* could aid in identification of metabolic bottlenecks as well as in elucidating the underlying reason for the significantly better performance of industrial strains compared to low producing strains.

## Results

### The RAVEN Toolbox

**Figure 1:**
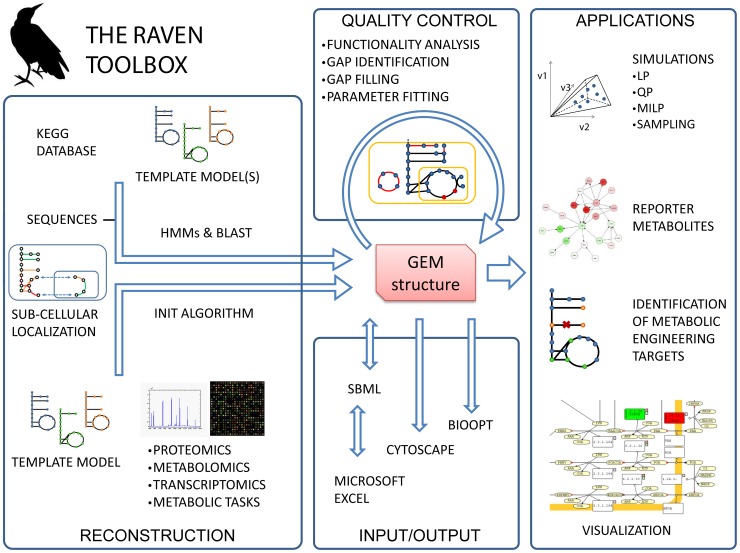
The RAVEN Toolbox. The software allows for reconstruction of GEMs based on template models or on the KEGG database. The resulting models can be exported to a number of formats, or they can be used for various types of simulations. The RAVEN Toolbox has a strong focus on quality control. Visualization of simulation result and/or integration of other types of data can be performed by overlaying information on pre-drawn metabolic maps. The software also implements the INIT algorithm, which is a powerful approach for reconstruction of tissue-specific models [59]. HMM: Hidden Markov model, LP: Linear programming, QP: Quadratic programming, MILP: Mixed-integer linear programming.

A software suite named the RAVEN Toolbox (Reconstruction, Analysis, and Visualization of Metabolic Networks) was developed. The toolbox is a complete environment for reconstruction, analysis, simulation, and visualization of GEMs and runs within MATLAB. The software imports and exports models in two formats: the widely used Systems Biology Markup Language (SBML) format [25] and a Microsoft Excel model representation. Both these formats allow for extensive annotation of model components, such as International Chemical Identifier strings (InChI) [26] for metabolites or database identifiers for reactions and genes. The native model format for the RAVEN Toolbox follows the format of the yeast consensus metabolic network [27], but models in the COBRA Toolbox format can also be imported [21]. The Microsoft Excel representation enables the user to set simulation parameters such as bounds and objective function coefficients directly in the spread sheets. This simplifies the modeling process for users not comfortable with working within a scripting environment, as well as providing a simpler, but less rigorous, model format compared to SBML. The software, together with a manual, a set of tutorials and a detailed description of the supported file formats is available through the BioMet Toolbox [28] (http://www.sysbio.se/BioMet). Figure 1 summarizes the capabilities of the RAVEN Toolbox.

The software has three main foci: 1) automatic reconstruction of GEMs based on protein homology, 2) network analysis, modeling and interpretation of simulation results, 3) visualization of GEMs using pre-drawn metabolic network maps.

#### Automated reconstruction of GEMs based on protein homology

Previously published GEMs represent a solid basis for metabolic reconstruction of models for new organisms, in particular if the organisms are closely related and therefore share many metabolic capabilities. The main advantage of using existing models compared to reaction databases, such as KEGG or BRENDA [29], is that they contain information that can be difficult to obtain in an automated manner, in particular directionality and compartmentalization. There have been attempts to predict the directionality of reactions based on the estimates of the standard Gibbs energies of formation for the involved metabolites [30]. However, we believe that manually reconstructed networks for related species can be a more reliable source of directionality information. The same is true for compartmentalization. Even though the RAVEN Toolbox contains methods for inferring subcellular localization based on predictors, it is to be viewed as an aid rather than an exact method. GEMs are also typically constructed for modeling purposes, which is not the case for reaction databases. The downside is that only reactions present in the template models can be included. The RAVEN Toolbox therefore contains two approaches for automatic generation of draft models; while the method mentioned above relies on the metabolic functions represented in previously published models, the complementary method uses the KEGG database for automatic identification of new metabolic functions that are not included in the published models.

The first approach lets the user supply a number of existing GEMs and FASTA files with protein sequences for the template models and for the organism of interest. The software then generates a draft model based on protein orthology. The default implementation uses bi-directional BLASTp [31] for evaluation of protein homology, but the software also supports other homology measurements as long as a score can be assigned to each pair wise protein comparison. The resulting model can be exported as a SBML file or be used in MATLAB for simulation and further analysis.

The second approach is also based on protein homology but requires no template models. Instead it relies on the information on protein sequences and on the assigned metabolic reactions that is available in the KEGG database. The method makes use of the KEGG Orthology (KO) IDs, which are manually annotated sets of genes that encode some specified metabolic function. Each KO is associated with a number of metabolic reactions. The aim of the present method is to assign genes to these KOs based on the consensus protein sequence. The tool first downloads all relevant parts of the KEGG database to a local directory and parses these files to generate a GEM representing a metabolic network across all of the species annotated in KEGG, i.e. this would lead to a network comprising 7029 metabolites, 8398 reactions and 843369 genes, when using the most current version of the KEGG database. A GEM for the organism of interest is then constructed by choosing a subset of this larger model and linking the reactions with the corresponding genes. The protein sequences for each KO are retrieved and aligned using MUSCLE [32]. The user has the option to only use genes from organisms of a given phylogenetic distance from the target organism, e.g. only fungal genes or only eukaryotic genes. A hidden Markov model is then generated based on the sequences for each KO using HMMER [33]. The final step is the querying of the set of HMMs with the protein sequences of the organism of interest. If a gene has a significant match to one KO, the reactions associated to that KO are added to the model together with the corresponding gene. This process is fully automated, and the user only needs to supply a FASTA file with protein sequences. Users who do not subscribe to KEGG can download pre-trained HMMs for eukaryotes and prokaryotes through the BioMet Toolbox (http://www.sysbio.se/BioMet). These HMMs are based on the last open version of KEGG. More advanced users can set parameters that affect how genes are mapped to KOs and how general, unbalanced, or otherwise problematic reactions from KEGG should be dealt with.

#### Model analysis and simulation

The approach proposed above will facilitate and accelerate the generation of a draft metabolic network reconstruction. The automated reconstruction can lead to some loss of control compared to a stricter manual, bottom-up approach. It is therefore important to identify and fill gaps in the model to ensure that the network is functioning as required. In a high quality model all reactions should be able to have a flux if all uptake and excretion reactions are allowed and net synthesis of most metabolites should be possible (the exception would normally be some co-factors). The second criterion is important, since the large degree of freedom in GEMs allow for internal loops where reactions can carry flux but where no net consumption or synthesis of metabolites occurs. The RAVEN Toolbox contains a number of methods to support the gap filling process. The following section describes the suggested workflow for gap identification and filling when starting from a draft network.

**Figure 2:**
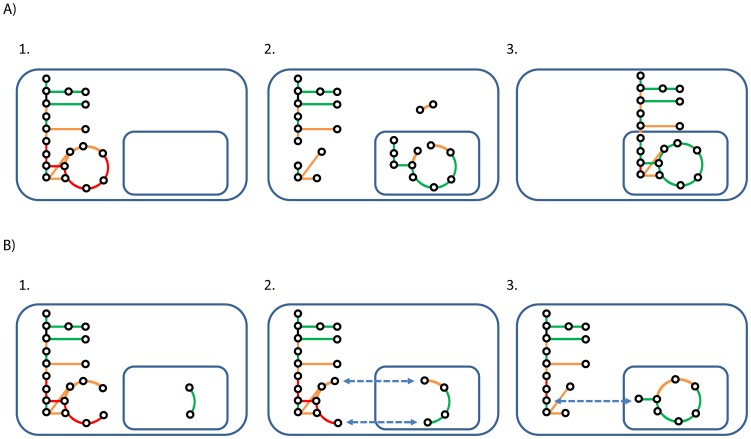
Prediction of subcellular localization of reactions. Circles correspond to metabolites and lines correspond to reactions. Green lines are reactions which are in their correct compartment according to the predictions. Red are reactions which are in an incorrect compartment and orange are reactions where there is no strong indication for either compartment. A) There is a tradeoff between connectivity and agreement with predicted localization. Network 1 represents the extreme case where connectivity is much more important than predicted localization scores. All reactions are then localized to the cytosol. Network 2 represents the other extreme case where the reactions are localized only based on localization scores and with no regard for connectivity. This would result in an unconnected network. Network 3 represents the case where the network is connected, while still being in good agreement with the localization scores. The underlying assumption in the algorithm is that a good network is characterized by being fully connected, in the sense that all metabolites are synthesized in at least one reaction and consumed in at least one reaction, while still being in good agreement with the localization scores and relying on the smallest possible number of transport reactions to achieve this. B) Summary of the localization algorithm. 1. The algorithm first randomly moves one gene product and its associated reaction(s) to another compartment. The probabilities depend on the scores for the gene products in their respective compartments. 2. This may result in an unconnected network. The algorithm then tries to find a small set of reactions which, when moved, reconnects the network. If moving these reactions would result in a large decrease of fitness, then the network is connected by including transport reactions for some metabolites instead. 3. The connected network is then scored as the sum of scores for all genes in their assigned compartment, minus the cost of all transport reactions that had to be included in order to keep the network connected. The user can set the relative weight given to transport compared to gene localization. The overall problem is solved using simulated annealing.

- Gap filling traditionally centers on adding reactions in order to enable production of all precursors needed for biomass production. However, it is equally important to ensure that the model cannot produce anything when there is no uptake of metabolites. The reactions which enable this type of behavior are typically those which involve polymers, metabolite pools, or other abstract metabolites but they can also simply be erroneous reactions. A brute force solution would be to exclude all reactions which are not elementally balanced, but this could result in a large fraction of the network being deleted, as many metabolites typically lack information about elemental composition. The *makeSomething* and *consumeSomething* functions identifies such reactions by solving the mixed integer linear programming (MILP) problem of finding the smallest set of reactions which results in the net synthesis or consumption of any metabolite. The solutions can then be cross-referenced to balancing information from *getElementalBalance* in order to identify reactions which are both active and have wrong/lacking composition. This process can also be done automatically using *removeBadRxns*.
- After the user has added relevant exchange reactions *canProduce*/*canConsume* can be used to generate a list of the metabolites that can have net synthesis or consumption. Early on in the reconstruction process it is likely that not all biomass precursors can be synthesized. The function *checkProduction* can be very useful in this situation. It calculates the smallest set of metabolites which must have net synthesis in order to enable net synthesis of all other metabolites. This gives the user information such as “in order to synthesize biomass, you must enable synthesis of valine and coenzyme A” or “if synthesis of choline is enabled, the following set of metabolites could also be synthesized”. The function also allows the user to set rules about merging compartments, since it can be easier to first make sure that the model is functional with merged compartments and deal with transport and sub-cellular localization afterwards.
- Ideally all reactions should be able to carry flux if all relevant exchange reactions are available. The function *haveFlux* can be used to identify reactions which cannot carry flux, and also to distinguish between reactions which cannot carry flux because some substrate cannot be synthesized and those which cannot carry flux because some product cannot be further consumed. However, because of the many internal loops in GEMs it is common that reactions can carry flux and appear well-connected even if they are not connected to the rest of the metabolic network. *getAllSubGraphs* can be used to identify such subnetworks using Tarjan's algorithm [34].
- The function *fillGaps* can be used to retrieve reactions from a set of template models or from KEGG in order to generate a functional network. The user can set constraints on their model, such as that it should be able to produce biomass from minimal media, and *fillGaps* will then solve the MILP problem of including the minimal set of reactions from a set of template models in order to satisfy the constraints. The same function can be used to enable net synthesis of all metabolites or to enable flux through all reactions. This approach is similar to that taken in Model SEED, and enables fully automatic model reconstruction. However, we suggest that GEM reconstruction should be done iteratively and with manual input and that the results from these algorithms are to be viewed as suggestions to point the user in the right direction.
- In eukaryotes the enzymatic reactions are distributed between different organelles. To determine which reactions occur where is a difficult task, and one of the more time-consuming steps in the reconstruction process. The RAVEN Toolbox takes a first step towards speeding up this step by including a method for assigning subcellular localization to enzymatic reactions in an automated fashion. The algorithm aims at assigning localization in a manner that is consistent with signal peptide composition and physiochemical protein properties, while at the same time maintaining a well-connected and functional network. The default predictor is WoLF PSORT, which is distributed with the RAVEN Toolbox [35]. A parser for other predictors, such as CELLO is also included [36]. In short, the algorithm works by generating fully connected solutions, which are then scored based on the agreement to the predicted localization and the number of transport reactions which had to be included in order to have a connected network. The problem is solved using simulated annealing. A more detailed description is available in Figure 2.

The RAVEN Toolbox also contains a number of methods for performing simulations using GEMs. In this aspect it is similar to the COBRA Toolbox [21]. Most of the features of COBRA Toolbox are also present in the RAVEN Toolbox, with the exception of dynamic FBA. This includes linear programming such as FBA, quadratic programming such as MoMA, mixed integer linear programming applications, and random sampling. Utility functions such as setting constraints and objectives, adding or removing model elements, presenting simulation outputs, sensitivity analysis, screening for gene deletions, and fitting model parameters such as maintenance ATP consumption are also included. For a full description of all functions of the toolbox, see the supplied manual. The RAVEN Toolbox uses MOSEK (MOSEK ApS, Copenhagen, Denmark) for solving the underlying optimization problems. MOSEK is proprietary software but a full featured license is freely available for academic use.

#### Validation of the workflow

The RAVEN Toolbox pipeline was validated by constructing a model for *Saccharomyces cerevisiae*, a model organism for which several GEMs have been constructed. To compare the quality of the automatically generated model to a manually curated one, some kind of reference was needed. As all models contain errors it would not be very relevant to simply compare the similarity between the RAVEN Toolbox generated model and a previously published model. Saccharomyces Genome Database (SGD) was therefore used as a reference with respect to the enzymes present in *S. cerevisiae* and their subcellular localization. A model was generated from KEGG in a fully automatic manner and then compared to the iIN800 model, a model which has been shown to have excellent simulation capabilities [37]. It should be noted that this fully automatic reconstruction is not how the RAVEN Toolbox is intended to be used for reconstruction. We suggest that the user view the output of each step as suggestions, and manually fill gaps or fix problematic reactions. However, we wanted to perform an evaluation of the overall reconstruction feature of the RAVEN Toolbox.

The model was generated using *getKEGGModelForOrganism* with the settings to only use eukaryotic genes when training the HMMs, a cutoff of 1e-30 when matching genes to the HMMs, and to exclude reactions labeled as general or incomplete in KEGG. *S. cerevisiae* genes were excluded in the training of the HMMs to simulate reconstruction of an organism for which there is little previous gene annotation. Not all unbalanced or erroneous reactions were labeled as such, and this resulted in that the KEGG model could produce some metabolites without any uptakes. *removeBadRxns* identified 79 reactions which enabled such production (see Table S1). Out of these 72 were unbalanced, general or polymer reactions and as such were correctly removed. 7 reactions were correct, but lacked composition about the metabolites (it is a setting in *removeBadRxns* whether it is allowed to remove such reactions).

Based on experimental minimal media the model was allowed uptake of glucose, phosphate, sulfate, NH3, oxygen and the essential nutrients 4-aminobenzoate, riboflavin, thiamine, biotin, folate, and nicotinate [38]. Uptake of the carriers carnitine and acyl-carrier protein was allowed for modeling purposes (many compounds are bound to them and therefore net synthesis of these compounds is not possible without them). This was the only manual step in the reconstruction of the yeast model.

**Figure 3:**
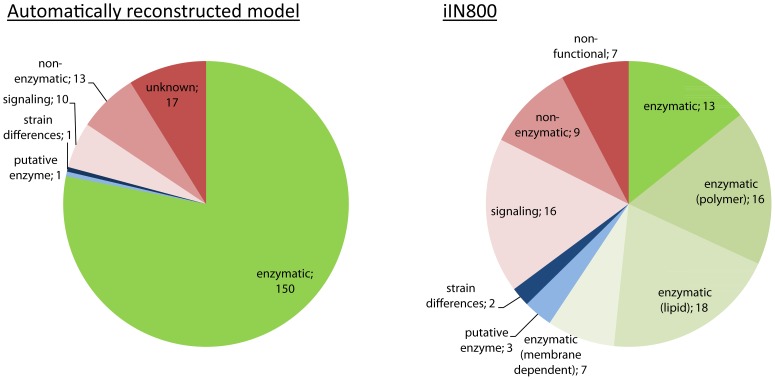
Overview of the genes which are unique to the automatically reconstructed model and iIN800, respectively. Saccharomyces Genome Database was used to classify the genes. Green corresponds to genes where the function is well-defined and suited for GEMs, basically enzymes involved in metabolism. Red corresponds to genes where the function is unknown, where the corresponding protein is not an enzyme or where the function is in signaling rather than metabolism. These genes should normally not be present in a GEM. Blue corresponds to genes that are putative enzymes or where the ORF is a functional enzyme in some strains but not in others. As can be seen, the automatically reconstructed model has both a larger number of unique genes and a larger proportion of enzymes compared to the published model. For iIN800 some enzymatic genes are further classified as “polymer”, “lipid” or “membrane”. These are parts of metabolism where an automatically generated model from KEGG would have particular drawbacks compared to a manually reconstructed model. “Polymer” corresponds mainly to genes involved in sugar polymer metabolism, which is an area that contains many unbalanced reactions in KEGG. Such reactions were excluded when the validation model was generated, so the corresponding genes could not be included. The same holds for “lipid”, where the reactions contain many general metabolites. This also results in excluded reactions. “Membrane” corresponds to reactions which depend on one metabolite but in two different compartments. This compartmentalization information is absent in KEGG so the equation becomes incorrect and it is therefore excluded.

The resulting model contained 1126 reactions, 1144 metabolites and 713 genes (before compartmentalization). 521 (73%) of those genes were shared with iIN800. 192 genes were unique to the automatically reconstructed model and 91 genes were unique to the iIN800 model (since there are no transport reactions in KEGG, all transporters were excluded from iIN800 for the purpose of this comparison). Figure 3 shows a classification of the genes that are unique to either the automatically reconstructed model or to iIN800 (see Table S2 for details). As can be seen, the automatically reconstructed model has a significantly larger proportion of enzymes compared to the published model.

Given the inputs the model could have net-synthesis of 476 (42%) of the 1144 metabolites (see Table S3 for details). As a comparison, 456 (66%) out of 683 unique metabolites in iIN800 could be synthesized given the same inputs. Among the 476 metabolites were 19 out of the 22 standard amino acids (leucine, methionine, and taurine could not be synthesized), the nucleotides needed for RNA and DNA synthesis (ATP, GTP, CTP, UTP, dATP, dGTP, dCTP, and dTTP), fatty acids, sterols such as lanosterol and ergosterol, important co-factors such as NADH, NADPH, FADH2 and CoA, and the building blocks needed for cell wall assembly (UDP-glucose, UDP-N-acetyl-D-glucosamine and mannose). The only major biomass constituents that could not be synthesized were complex lipid compounds such as phospholipids and sphingolipids. This is because of the combinatorial nature of fatty acid metabolism (given ∼20 fatty acids there are 20!/2!(20-2) = 190 possible versions of phosphatidylcholine) and how it is represented in KEGG.

As the next step of the fully automatic reconstruction, *fillGaps* was used to automatically fill gaps in the yeast network using the full KEGG database as a template. This resulted in 45 reactions being added, which in turn enabled the synthesis of 91 metabolites that could previously not be synthesized (see Table S4). Among them were the three amino acids that were previously missing. A closer investigation of the reactions which were added (see Table S5) showed that out of the 45 added reactions, 17 had evidence to support that they should be included in the model, 9 had inconclusive or missing evidence, and 19 reactions should not have been included in the model. 5 of the 91 genes that were previously unique to iIN800 were also added in this process.

The RAVEN Toolbox also contains a method for partitioning enzymatic reactions to compartments in a manner that keeps the network connected, but at the same time in agreement with the results from predictors of protein localization (see Figure 2 for details). The default predictor, WoLF PSORT, was used to predict the protein localization of all ORFs in the FASTA file. *predictLocalization* was then used to partition the network between mitochondria and cytosol. The transport cost was set to 0.1. Table S6 lists the genes for which the corresponding reactions were assigned to the mitochondria. 119 gene products were assigned to the mitochondria and the remaining 594 gene products were assigned to the cytosol. Out of the 119 predicted mitochondrial gene products, 72% were listed as mitochondrial in the SGD based on experimental evidence. The same calculations for iIN800 give that 91 gene products are mitochondrial and that 83% were listed as mitochondrial in SGD. Localization predictions based only on primary protein sequences are not very exact, and the resulting model from *predictLocalization* will not be totally biologically correct. The main issue is that all transport reactions are formulated as passive diffusion, while in reality other types of transport are also taking place. However, the method is able to quickly generate a connected model where the enzyme localizations are in almost as good agreement to SGD as a published model. This could be useful for many applications, such as when using metabolic networks for integrating omics data, and it constitutes a first step towards fully automated reconstruction of eukaryote GEMs.

These results show both strengths and weaknesses of using a fully automatic approach to reconstruction. A model capable of producing all the needed building blocks for synthesis of protein, RNA, DNA, and the cell wall was generated solely from a FASTA file and with almost no manual input. The automated gap filling identified 17 new reactions, out of which 8 were not present in the published *S. cerevisiae* model. As was shown in Figure 3, the quality in terms of included genes was as good or better compared to the published model. On the other hand, the gap filling included 19 reactions which did not belong in the model, and complex lipids could not be synthesized. The sub-cellular localization of enzymes was up to par with the published model, but with the drawback that all transport reactions were formulated as passive diffusion. In a real situation a reconstruction should therefore be done in an iterative manner, with manual input after each iteration (the user would, for example, remove the 19 bad reactions from the template model and then run *fillGaps* again).

#### Visualization of GEMs

**Figure 4:**
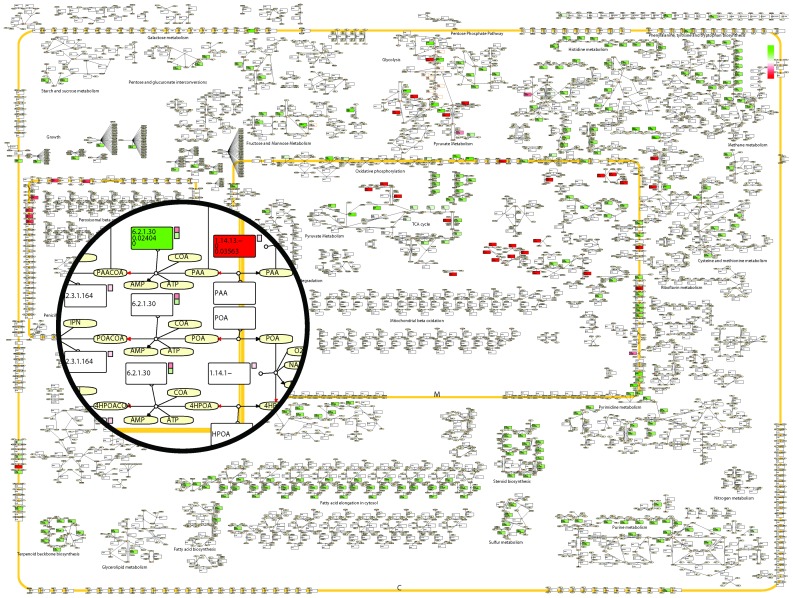
Example of the visualization capabilities of the RAVEN Toolbox. The figure shows a small section of the *Penicillium* metabolic map, depicting peroxisomal penicillin metabolism, superimposed on the full map. Rectangles correspond to reactions and ellipses correspond to metabolites. The broad yellow line represents the peroxisomal membrane. Reactions are colored based on the log-fold change in flux between a reference and a test case, where green represents a higher flux in the test case and red a lower flux. The positive direction of reversible reactions (defined as from left to right in the model equations) is indicated by a red arrow head. For reactions carrying flux in any of the simulated cases, the flux values are printed in the reaction box. The small squares to the right of some of the reactions correspond to the log-fold change of transcript levels of the genes associated to that reaction. The gene-reaction relation is retrieved from the model structure and not implicitly specified in the CellDesigner map.

Stoichiometric metabolic models have been proven to generate remarkably good predictions when it comes to the central carbon metabolism in microorganisms. However, the lack of kinetic and regulatory information is a rather large simplification and it is possible to get simulation results that have little biological meaning (such as thermodynamically disallowed loops). It is therefore imperative to understand the underlying reasons for a change in predicted phenotype after a perturbation such as a gene deletion. Due to the large dimensionality of GEMs interpretation of flux distributions is a rather daunting task. Visualization of fluxes can aid with interpretation, as well as provide an instant overview of how the system functions. Software that aims at network visualization based purely on connectivity, such as Cytoscape [39] or CellDesigner [40], cannot provide a comprehensible or well organized image of GEMs due to their size. The RAVEN Toolbox allows for visualization of simulation results based on manually drawn maps. The maps are drawn in CellDesigner and each reaction is labeled with the corresponding reaction identifier in the model. The map can then be imported to MATLAB and the reactions are colored according to the change in the corresponding flux between simulation conditions. Gene expression data can be incorporated in the map to illustrate the correlation between flux and gene expression. This will extraordinarily facilitate the comparison and interpretation of flux distributions found for different environmental conditions. The resulting map is exported as a pdf-file. Figure 4 show a close up on penicillin metabolism in the peroxisome overlaid on the full *P. chrysogenum* map (see following section for details).

### Comparative genomics of template species

In order to assign metabolic functions to the genes present in the *P. chrysogenum* genome, sequence alignment analysis was performed. Three fungi from the *Aspergillus* genus (*A. oryzae*, *A. niger* and *A. nidulans*) were selected for sequence comparison based on being closely related fungi outside of the *Penicillium* genus and on having previously reconstructed GEMs (see Figure S1). Table 2 shows some genome characteristics of the *Aspergilli* in comparison with *P. chrysogenum*. Initially pairwise comparison was done by similarity searching of the protein sequences of *P. chrysogenum* against the protein sequences known to be involved in the metabolism of the three *Aspergillus* species as described in the Methods section. With a chosen threshold of the E-value, identity, and alignment length, a list of inferred metabolic functions was generated. The results are summarized in Table 2. Pairwise comparison shows that *A. oryzae* has the highest number of sequence homologues of proteins with metabolic functions with *P. chrysogenum* (915 sequences). This result suggests that metabolism of *A. oryzae* is probably closer related to *P. chrysogenum* than *A. nidulans* and *A. niger* which have less sequence homologues of 576 and 563, respectively. Upon completion of the similarity searching, the results suggest that 1143 genes in *P. chrysogenum* could be assigned as orthologous metabolic genes from the three *Aspergillus* species used for comparison. The large number of metabolic orthologues indicates that the existing GEMs for closely related species could be a sound foundation upon which to reconstruct the new model.

**Table 2:** Comparison of genome characteristics and metabolic function assignment between *P. chrysogenum* and three *Aspergillus* species.

Features	*ANi*	*AO*	*AN*	*PC*
Genome size (Mb)	30.1	37.2	34.9	32.2
Number of chromosomes/supercontigs	8	8	8	49
Number of total protein sequences	10 560	12 074	11 197	12 811

Functional assignments				
Pairwise comparison	*ANi* and *PC*	*AO* and *PC*	*AN* and *PC*	*PC*
Number of protein sequence orthologues^a^	5749	5614	5632	-
Number of metabolic orthologues based on COG^b^	1316	1471	1313	2330
Number of metabolic orthologues based on GEMs^c^	576	915	563	1143

*ANi*: *A. nidulans*, *AO*: *A. oryzae*, *AN*: *A. niger*, *PC*: *P. chrysogenum*.

a Proteins were regarded as orthologues if E-value <1e-30, identity >40%, sequence coverage >50%, and alignment length >200 amino acids.

b Described as being present in the functional category of metabolism based on the COG database [61].

c Described as being present in the corresponding previously published genome-scale metabolic model; *A. nidulans iHD666* [52]; *A. niger iMA871* [53]; *A. oryzae iWV1314* [54].

### Reconstruction and comparative analysis of the *P. chrysogenum* metabolic network

Using the RAVEN Toolbox the metabolic network of *P. chrysogenum* metabolic network was reconstructed. The metabolic network comprises 1471 unique metabolic reactions in four sub-cellular compartments; extracellular, cytosolic, mitochondrial, and peroxisomal (Table 3). 1006 ORFs are associated to the reactions, 89 of which participate in one of 35 protein complexes. In parallel to the automatic reconstruction, an extensive literature study was performed. In total 440 cited articles provide experimental evidence for the majority of the reactions. All model components were extensively annotated to adhere to the MIRIAM standard for biological models [41]. The model was validated with respect to 76 important metabolic functions (the supplementary file simulations.xls is an input file to *checkTasks*, which was used to performed the validation). There are 30 reactions in the model which cannot carry flux if all uptakes are allowed, i.e. dead-end reactions (see Table S7). According to the naming conventions for metabolic networks the presented model is denoted as *iAL1006* [42]. Table 3 shows the division of model elements between the four compartments.

**Table 3:** Network characteristics of the reconstructed metabolic network of *P. chrysogenum*.

ORFs	1006
EC-numbers	627
Metabolites^a^	1235
Extracellular metabolites	160
Cytosolic metabolites	728
Mitochondrial metabolites	242
Peroxisomal metabolites	105
Reactions^b^	1471
Extracellular reactions	175
Cytosolic reactions	835
Mitochondrial reactions	324
Peroxisomal reactions	137

a Exchange metabolites are not included.

b Exchange reactions are not included. Transport reactions from the cytosol to any other compartment are included in the count for that compartment, i.e. mitochondrial transport reactions are regarded as mitochondrial reactions.

**Figure 5:**
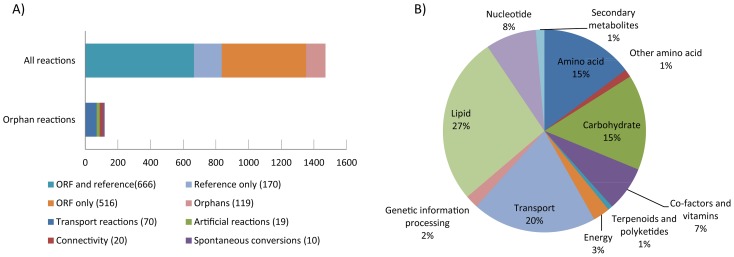
Evidence level for the *P. chrysogenum* metabolic network. A) Properties of the reconstructed network. The top bar shows the support for the 1471 unique reactions (not counting exchange reactions) sorted by the type of evidence. The bottom bar shows the orphan reactions; reactions inferred without supporting ORFs or literature references. B) ORF classification. The ORFs in the model are classified into broad groups based on KEGG classification.

Figure 5 summarizes the literature support for the reactions in the model and shows a classification of the ORFs in the model based on the KEGG pathways. The full list of reactions, metabolites, and genes are supplied in Microsoft Excel format and SBML format in Dataset S1. Both these formats are compatible with the RAVEN Toolbox. To illustrate the metabolic network, and to aid in interpretation of gene expression data and simulation results, a map of the full model was drawn in CellDesigner and annotated so as to be compatible with the visualization functions in the RAVEN Toolbox. The CellDesigner file is available in Dataset S1. Even though the map is drawn for *Penicillium* metabolism it can be used as a template for generation of maps for other organisms as well. Lastly, the *P. chrysogenum* GEM has also been added to the model repository in the BioMet Toolbox, which allows for a variety of analyses and simulations to be carried out.

### Comparison of fungal metabolic networks

**Figure 6:**
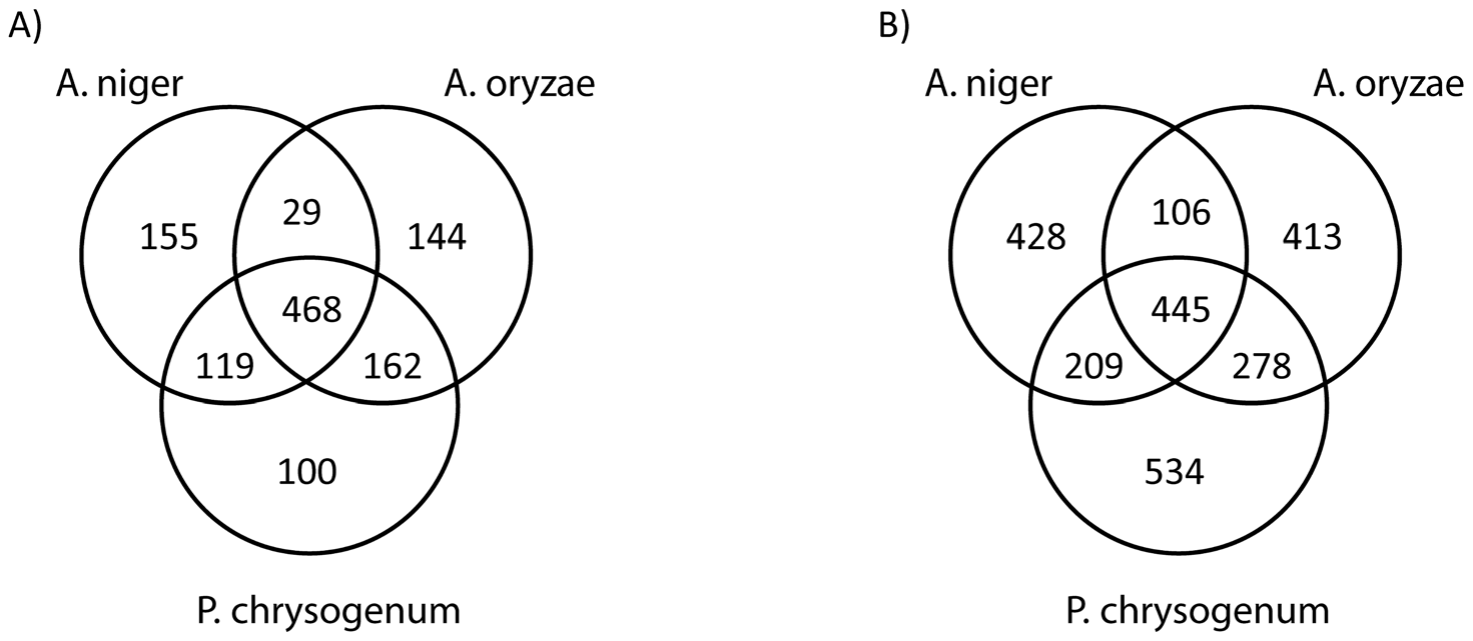
Venn diagrams of model statistics for the template models *A. oryzae* iWV1314 and *A. niger* iMA871 and the *P. chrysogenum* iAL1006 model. A) The number of chemically distinct metabolites shared and specific for the three models, not counting presence in multiple compartments. B) The number of unique reactions shared and specific for the three models. The overlap with *A. nidulans iHD666* is not shown here.

To evaluate the similarity between the reconstructed network and the template networks, the networks were compared with respect to identical reactions and involved metabolites. Only *A. oryzae iWV1314* and *A. niger iMA871* were used in the comparison since only a small number of reaction were inferred from *A. nidulans iHD666*. Figure 6 illustrates the results. 534 reactions were unique to *iAL1006*. The large discrepancy between the models is primarily because of differences in how lipid metabolism is formulated and due to differences in localization. There are also differences in how reactions catalyzed by protein complexes are described, where one reaction for each subunit is formulated rather than lumping the reactions. The difference in metabolic capabilities between the models is therefore smaller than what is indicated by the Venn diagrams (Figure 6). The unique capabilities of the *P. chrysogenum* model are mainly in penicillin metabolism and transport (data not shown). In general, the reactions that were inferred from *A. oryzae* but not from *A. niger* are predominantly involved in co-factor synthesis and in sugar polymer metabolism. The reactions inferred from *A. niger iMA871* but not from *A. oryzae iWV1314* are mainly involved in lipid metabolism. The key statistics of the reconstructed *P. chrysogenum* network compared to those of other fungal networks is available in Table S8.

### Biomass composition and parameter fitting

Growth is described as production of biomass, which in turn is regarded as drain of the macromolecules and building blocks that constitute the cellular components. The demand of each component is estimated based on published data on the biomass composition. The main components and their content within the biomass are listed in Table 4 (see also Table S9 for a detailed description). The cost of biomass production does not only include synthesis of precursors and polymerization of macromolecules, but also factors such as maintaining turgor pressure, transport costs, protein turnover, and membrane leakage. These costs are summarized as an ATP requirement for non-growth associated maintenance, m_ATP_, and for growth associated maintenance, K_xATP_, i.e. ATP costs not directly associated with biomass synthesis but associated with cell growth (can be maintenance of membrane potentials across an expanding cell). These parameters were determined by linear regression to glucose-limited chemostat experiments in the presence of phenoxyacetic acid (POA) [43]. The values were hereby estimated to be 4.14 mmol ATP/g DW/h for m_ATP_ and 104 mmol ATP/g DW for K_xATP_. The growth-associated ATP cost is significantly higher than for the template organisms (64 mmol ATP/g DW/h in *A. niger iMA871*). This could possibly be an effect of the presence of phenoxyacetic acid, which is added to the fermentation medium under industrial penicillin producing conditions. It is believed that phenoxyacetic acid, being a lipophilic weak acid, acts as a proton un-coupler which would manifest itself as a high ATP maintenance cost [44]. The P/O ratio is fitted by assigning the number of cytosolic protons needed to synthesize one ATP by the F_0_F_1_-ATPase. This is a small simplification since the number of protons pumped across the mitochondrial membrane might also differ between organisms. This parameter was estimated to 3.75 (3.88 in *A. niger iMA871*). Figure S2 shows the agreement of model simulations with experimental fermentation data after parameter fitting.

**Table 4:** Biomass composition of *P. chrysogenum*.

Components	Content (g/g DW)
Protein	0.45
RNA	0.08
DNA	0.01
Lipids	0.05
Phospholipids	0.035
Sterolesters	0.010
Triacylglycerides	0.005
Carbohydrates	0.25
Cell wall	0.22
Glycogen	0.03
Soluble pool	0.08
Amino acids	0.04
Nucleotides	0.02
Total^a^	0.90

a 8% of the dry weight is constituted by ash [43]. The remaining 2% are other soluble metabolites.

### Simulations and integrative data analysis of penicillin biosynthesis

A genome-scale metabolic model is a powerful tool that can be used for exploring the metabolic capabilities of the cell, as well as being used as a scaffold for integrative data analysis. Here we present two case studies to illustrate the use of the reconstructed *P. chrysogenum* model. The first case is a study of penicillin yields and in particular the relative importance of ATP and NADPH provision during penicillin production. In the second study we show how the model can be used to integrate fermentation data with transcriptome data using a recently published sampling algorithm to aid in the interpretation of high-throughput data [4].

#### Penicillin yields

Penicillin production is associated with an increased requirement of energy in the form of ATP; in the condensation of the three precursor amino acids to form the tripeptide ACV; in the reduction of sulfate; and when a side chain (the precursor molecule which is supplied to the media and which differs depending on the type of penicillin produced) is activated by ligation to coenzyme A. Penicillin production is also associated with a large requirement of NADPH; primarily needed for the reduction of sulfate but also in the biosynthesis of valine and homoserine from α-ketobutyrate. Elucidating the impact increased ATP requirements have compared to the NADPH requirements is useful when choosing among possible metabolic engineering strategies.

Different types of penicillin can be produced by changing the side chain that is supplied to the medium (e.g. supplementation of phenylacetic acid result in penicillin G and supplementation of phenoxyacetic acid result in penicillin V). However, this has no impact on the yield and it is therefore not necessary to specify the type of penicillin being produced for theoretical evaluations. The maximum theoretical yield of penicillin on glucose with sulfate as the sulfur source was calculated to be 0.42 mol penicillin/mol glucose using the reconstructed genome-scale metabolic model. This is in agreement with what has previously been published [45]. If the sulfur source is sulfite the maximal theoretical yield is found to be 0.45 mol penicillin/mol glucose and if it is hydrogen sulfide it is 0.51 mol penicillin/mol glucose. The difference between using sulfite and hydrogen sulfide is relatively large and can be attributed to the differences in NADPH cost (3 NADPH are consumed in the sulfite reduction to hydrogen sulfide). This points to the importance of NADPH availability for penicillin production. To investigate the effect of ATP an artificial reaction was included that allowed for ATP production from ADP without any energetic costs. This resulted in a yield of 0.52 mol penicillin/mol glucose, using sulfate as the sulfur source. The conclusion is that ATP availability has a relatively small effect on the yield, comparable to that of NADPH consumption in the sulfate reduction. The shadow prices (how much the penicillin production can increase if the availability of a metabolite were to increase by a small amount) were calculated to be 0.015 mol penicillin/mol ATP, 0.040 mol penicillin/mol NADPH, and 0.037 mol penicillin/mol NADH.

NADPH and NADH are similar when it comes to energy content, but have different roles in the metabolism, where NADPH serves primarily anabolic roles and NADH primarily catabolic roles. NADPH is mainly produced in the pentose phosphate pathway, which makes NADPH somewhat more energetically expensive to regenerate compared to NADH. In order to investigate the relative importance of NADH and NADPH an artificial reaction was included that allowed for production of NADPH from NADH to simulate a potential increase of the NADPH availability. Simulations were then carried out maximizing first for growth and then for penicillin production. The resulting flux through the artificial reaction was 8.5 times larger when maximizing for penicillin than when maximizing for growth. This demonstrates that the cells will have a much higher NADPH demand at high penicillin yields compared to normal growth conditions. Redirecting a higher flux through the pentose phosphate pathway and/or introducing NADH-dependent versions of NADPH-consuming enzymes could therefore be potential metabolic engineering strategies for achieving higher penicillin yields.

For the direct identification of possible metabolic engineering targets a gene deletion analysis was performed by searching for sets of gene deletions that result in an increased yield of penicillin, and which would stoichiometrically couple penicillin production to growth. This was performed using FBA, and combinations of up to three gene deletions were evaluated (MoMA was also applied and gave similar results). The only targets which could be identified were the deletion of any of the genes responsible for breakdown of phenylacetic acid (homogentisate 1,2-dioxygenase, maleylacetoacetate isomerase, or fumarylacetoacetase). Deletion of any of these genes resulted in a 21% increase in penicillin production when maximizing for growth.

#### Identification of transcriptionally regulated metabolic bottlenecks

The metabolism of cells is redundant in the sense that different sets of metabolic reactions can be used to generate the same net phenotype. A recently developed method aims at finding potential metabolic engineering targets by identifying genes that are differentially expressed between different cultivation conditions and where the corresponding reactions exhibit significantly changed fluxes for the same conditions [4]. Changes in expression level of such genes are then assumed to be likely to result in altered fluxes. The algorithm finds these transcriptionally regulated reactions by random sampling of the solution space, after which it compares the statistics of the sampling with the statistic of the mRNA expression. Here we applied this method to compare the high producing industrial strain DS17690, which has been developed by DSM, and the low producing reference strain Wis 54-1255 (see Methods for details) [46].

**Figure 7:**
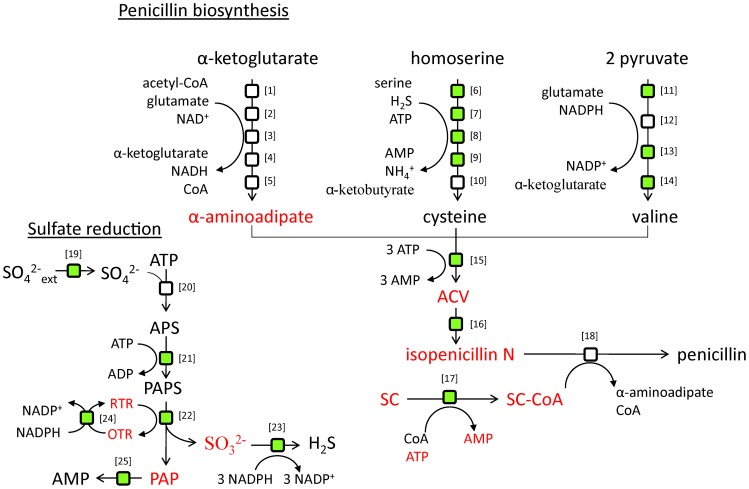
Integrative analysis of a high and a low producing strain. Depicts synthesis pathways of penicillin and important precursors. Green boxes correspond to reactions identified as being transcriptionally controlled and up-regulated by the algorithm (see text). Metabolites around which significant transcriptional changes occur compared to a low producing strain are colored red. SC: side chain (e. g. the precursor molecule phenylacetic acid). The biosynthesis of penicillin starts with the condensation of the three amino acids α-aminoadipate (an intermediate in the L-lysine biosynthesis pathway), L-cystein, and L-valine to form the tripeptide ACV. ACV is further converted to isopenicillin N. For the industrially relevant types of penicillin a side-chain is supplied to the media. This side-chain is activated by ligation to coenzyme A. In the last step of penicillin biosynthesis an acyl transferase exchanges the α-aminoadipate moiety of isopenicillin N with the side-chain, thereby generating penicillin and regenerating α-aminoadipate. Since L-cystein is a sulfur-containing amino acid penicillin production is also tightly associated with sulfur metabolism. The corresponding model IDs for the enzymes are indicated within parentheses. [1] homocitrate synthase (r0683); [2] homocitrate dehydrase (r0684); [3] homoaconitate hydrase (r0685); [4] homoisocitrate dehydrogenase (r0688); [5] α-aminoadipate aminotransferase (r0689); [6] homoserine transacetylase (r0600); [7] O-acetylhomoserine sulfhydrylase (r0601); [8] cystathione-β-synthase (r0632); [9] cystathione-γ-lyase (r0606); [10] acetate CoA ligase (r0025); [11] acetolactate synthase (r0465); [12] ketol-acid reductoisomerase (r0653); [13] dihydroxy acid dehydrase (r0656); [14] branched chain amino acid transferase (r0648); [15] ACV synthase (r0814); [16] isopenicillin N synthase (r0812); [17] acyl CoA ligase (side chain dependent, reaction is for phenylacetate CoA ligase) (r0747); [18] isopenicillin N N-acyltransferase (r0813); [19] sulfate permease (r1408); [20] sulfate adenyl transferase (r1151); [21] adenyl sulfate kinase (r1147); [22] phosphoadenyl sulfate reductase (r1148); [23] sulfite reductase (r1149); [24] thioredoxin reductase (r0419); [25] 3′(2′),5′-bisphosphate nucleotidase (r1150).

A total of 58 fluxes were found to be significantly changed between the high and low production strains (p<0.05) and 612 genes were differentially expressed (p<0.005). Out of those, 36 reactions were identified as having significantly higher flux and up-regulated genes (see Table S10), i.e. they are likely to have transcriptional regulation of their fluxes. Figure 7 shows some of the most important reactions in penicillin biosynthesis together with the responsible enzymes and the corresponding model IDs. Reactions that were identified as probably being transcriptionally controlled and up-regulated are highlighted. In addition, the Reporter Metabolites algorithm was used to identify metabolites around which significant transcriptional changes occurred [47]. These metabolites are highlighted in Figure 7 as well (see Table S11 for a full list of reporter metabolites).

As can be seen in Figure 7, a large proportion of the reactions identified as being a transcriptionally controlled are directly involved in penicillin metabolism (15 out of 38). This indicates that the capabilities of the industrial strain to produce penicillin to a large extent depend on the reactions closely related to penicillin metabolism, rather than more peripheral effects. Among these reactions are many of the reactions responsible for the synthesis of the amino acids that are precursors for ACV as well as the two penicillin producing reactions isopenicillin N synthase and ACV synthase, which is consistent with a study on the gene copy-number effect on penicillin production [48]. The phenylacetate:CoA ligase is high ranking but the acyl-CoA:isopenicillin N acyltransferase is absent, which is consistent with measurements of high activities of this enzyme and the low flux control estimated for this enzyme [49], [50]. Several of the reactions involved in sulfate reduction are present as well as the sulfate permease. It is interesting to note that none of the reactions in the pentose phosphate pathway are identified even though there is an increased demand for NADPH.

We also found that the pathway from α-ketobutyrate to succinate is identified to have both increased flux and increased gene expression. α-ketobutyrate is a by-product of cysteine production via the transsulfuration pathway, and it is used for isoleucine biosynthesis. Under normal growth conditions the demand for cysteine is less than that for isoleucine, meaning that all α-ketobutyrate is converted into isoleucine. However, during high-level penicillin production the cysteine production far exceeds the need for isoleucine, requiring an alternative route for α-ketobutyrate consumption. This route involves the decarboxylation of α-ketobutyrate to yield propionyl-CoA, which then goes into the methylcitrate pathway, eventually resulting in succinate [45]. Several of the reactions in this pathway are identified as transcriptionally controlled by the algorithm (2-methylcitrate synthase, 2-methylcitrate dehydratase, 2-methylisocitrate dehydratase, and methylisocitrate lyase). This finding strongly supports that the transsulfuration pathway is the dominating pathway for cysteine biosynthesis, even though the enzymes for the energetically more efficient direct sulfhydrylation pathway have been identified in *P. chrysogenum* [51].

## Discussion

The RAVEN Toolbox, a software suite for semi-automated reconstruction and simulation of genome-scale metabolic models was developed. The RAVEN Toolbox is the first software that contains methods both for model reconstruction and for a wide variety of simulation approaches. A visualization feature for simulation results and a feature that allows the user to manipulate metabolic models and set simulation parameters via Microsoft Excel are provided in order to make the software easy to use. The RAVEN Toolbox was evaluated for its ability to reconstruct GEMs by generating a model for *S. cerevisiae*. The reconstructed model compares well with a manually reconstructed model. This demonstrates that the RAVEN Toolbox is useful for reconstruction of novel models, in particular eukaryotic models, due to its feature for automatic assignment of sub-cellular localization. We used the RAVEN Toolbox to reconstruct a GEM for *P. chrysogenum* by using three models for closely related fungal species. Extensive manual validation of the model was performed; both to validate the reconstruction method and to ensure a high-quality model. The resulting *P. chrysogenum* model consists of 1471 reactions, 1235 metabolites and 1006 genes. 440 cited articles provide experimental evidence for the majority of the reactions. Considerable efforts were spent on standardizing and annotating the template models in order to adhere to MIRIAM standards. The standardized template models and the reconstructed *P. chrysogenum* model are available through the BioMet Toolbox. This collection of fungal models, together with the complementary method of generating metabolic networks based on the KEGG database, constitutes an excellent platform for the reconstruction of metabolic networks for other eukaryotic organisms.

## Methods

**Figure 8:**
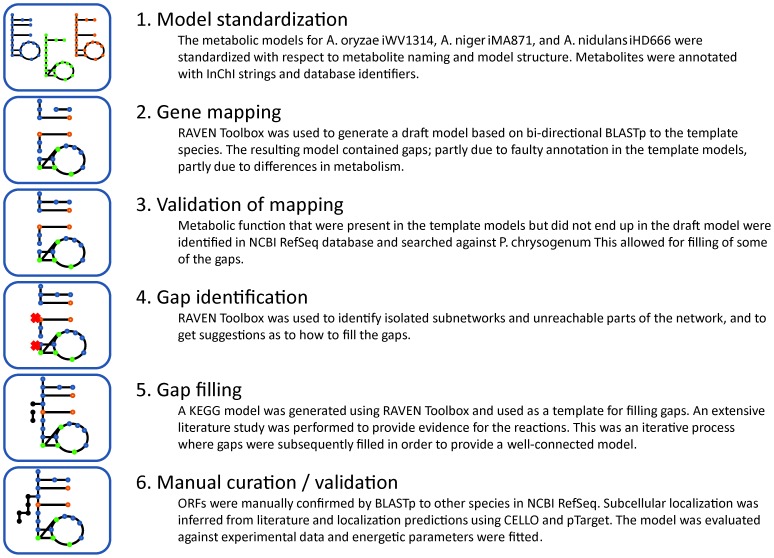
Overview of the iAL1006 reconstruction process.

The *P. chrysogenum* metabolic network was reconstructed based on a combination of automated reconstruction approaches, manual curation, and an extensive bibliomic survey. Figure 8 gives an overview of the whole reconstruction process.

### Inferring reactions based on protein homology

Three GEMs for other filamentous fungi, *A. nidulans* iHD666 [52], *A. niger iMA871* [53], and *A. oryzae iWV1314* [54], were used as template models for the reconstruction of a *P. chrysogenum* model. Efforts were taken in order to standardize the template models to facilitate the automatic reconstruction. This standardization primarily involved metabolite naming, but also how to represent more complex aspects of metabolism such as polymers and lipids. As part of the standardization effort a large majority of the metabolites were assigned database identifiers and chemical structure information. This annotation step allowed for verification that all reactions were elementally balanced, which in turn led to a number of inconsistencies in the template models being corrected. The revised models for the three *Aspergillus* species are available as up-dates in the BioMet Toolbox (http://www.sysbio.se/BioMet) [28].

A draft GEM for *P. chrysogenum* was then constructed based on bidirectional best hits of BLASTp between the template model proteins and their orthologues in *P. chrysogenum* using the RAVEN Toolbox. Proteins were regarded as orthologues if E-value <1e-30, identity >40%, sequence coverage (>50%) and alignment length (>200 amino acids).

The protein sequences of *P. chrysogenum* Wisconsin 54-1255 (annotation, version 1) were obtained from the EMBL database (http://www.ebi.ac.uk/embl/). The protein sequences of *A. nidulans* FGSC A4 (annotation, version 4) were taken from the Broad Institute database (http://www.broadinstitute.org/annotation/genome/aspergillus_group). The protein sequences of *A. oryzae* RIB40 (annotation, version 1) were taken from the DOGAN database (http://www.bio.nite.go.jp/dogan/project/view/AO). The protein sequences of *A. niger* ATCC1015 (annotation, version 3) were taken from the JGI database (http://genome.jgi-psf.org/Aspni5/Aspni5.home.html).

### Gap filling

The first draft model based on homology to template models contained gaps due to incorrect annotation in the template models and lacked reactions in parts of metabolism that were unique to *P. chrysogenum* (Figure 8). Therefore, another draft model was generated from KEGG using the RAVEN Toolbox. An E-value <1e-50 was used as cut off in the gene assignment. This model was used for filling gaps in the draft network and for suggesting metabolic pathways that were not included based on the template models. No reactions were included based solely on presence in the KEGG model, and gene assignments were only included after careful manual validation against the NCBI RefSeq database.

Gaps in the draft metabolic network were identified using the gap finding capabilities of the RAVEN Toolbox (Figure 8). The initial network was rather disconnected and biomass production from glucose was not possible. Firstly, the software was used to identify which metabolites had to be connected in order to produce biomass. This resulted in the addition of some spontaneous reactions from the template models. The second step was to ensure that as many metabolites as possible could be produced. When non-connected metabolites were identified, the KEGG model was queried for candidate reactions which could connect that metabolite. A targeted literature search was then conducted to find evidence for the presence of such a reaction. In only few cases this resulted in the addition of transport reactions based solely on connectivity issues (<2%, see Figure 5). The final step was to use the RAVEN Toolbox to identify reactions that could not carry a flux during growth on any of the available carbon sources. There are, however, sets of reactions that are included in the model even though they cannot currently carry a flux. One such example is the synthesis and loading of tRNA, which is included to allow for possible future extension of the model to cover protein synthesis.

### Compartmentalization and transport

The model has four compartments: extracellular space, cytosol, mitochondrion, and peroxisome. Reactions with unknown localization, or where the real localization is not represented by one of the compartments in the model, were assigned to the cytosol. Enzymes reported to be present in the cell wall were assigned to the extracellular space, and those present in the mitochondrial membrane were mainly assigned to the mitochondria. The peroxisome was included primarily because of its role in penicillin metabolism and β-oxidation of fatty acids. Transport reactions between compartments were inferred mainly from the template fungal models and backed up with literature evidence. However, there were situations where a transport reaction had to be included in order to have a functional network, even when no literature evidence could be found. For enzymes reported to have isoenzymes in several compartments, the ORF assignments to each compartment were based on localization predictions from CELLO [36] and pTarget [55].

### Simulations

Unless otherwise specified, simulations were carried out using unlimited uptake of oxygen, phosphate, sulfate, NH3, thiamin and pimelate. The carbon source was glucose. Excretion of all exchange metabolites was allowed. Biosynthesis of L-cysteine was only allowed through the transsulfuration pathway. Since the energy content of NADH and NADPH is similar it is possible that cycles convert one into the other. These cycles normally take the form of reversible reactions that can utilize either NADH or NADPH. Since such futile cycles make it difficult to study NADPH/NADH metabolism using GEMs, and since they are probably not active in the cell, they were identified and blocked in analogy to what has been done in previous models [10]. The way by which they were identified was minimizing/maximizing for the flux through an artificial reaction NADH+NADP(+)< = >NAD(+)+NADPH while not allowing for uptake of any carbon source. Reactions were then identified and deleted until this reaction could no longer carry a flux. The following reactions were deleted: D-mannitol:NAD+ 2-oxidoreductase (r0181); ethanol:NADP+ oxidoreductase (r0019); ethanol:NAD+ oxidoreductase (r0020); (S)-3-hydroxybutanoyl-CoA:NADP+ oxidoreductase (r0081). None of these reactions can be expected to be active during the studied conditions. Deletion of D-mannitol:NAD+ 2-oxidoreductase does not inactivate the NADPH regenerating mannitol cycle, which has a role in NADPH regeneration in many fungal species [56].

The penicillin yields were calculated by setting the glucose uptake rate to 1.0 mmol/gDW/h and maximizing for penicillin production. Free ATP was simulated by including an artificial reaction in the form ADP+Pi = >ATP+H2O.

### Integrative analysis

A random sampling algorithm was applied in order to identify transcriptionally regulated metabolic bottlenecks [4]. Flux data and gene expression levels for aerobic, glucose-limited chemostat fermentation of DS17690 and Wis 54-1255 were used as input to the algorithm [57]. The exchange fluxes were fitted to the reported values using a quadratic fitting. 5000 sampling iterations were performed for each of the two strains. The expression data set of the study were retrieved from GEO database (GSE9825) as CEL format then normalized together with PLIER workflow (http://media.affymetrix.com/support/technical/technotes/plier_technote.pdf). Two way ANOVA were employed to evaluate the differentially expressed genes with respect to the strain (DS17690 and Wis 54-1255) with multiple correction following [58]. The Reporter algorithm [47] was employed to integrate the transcriptome data with the reconstructed GEM to identify key metabolites in the network.

## Supporting Information

Dataset S1 The iAL1006 genome-scale model of *P. chrysogenum* in SBML and Excel formats, together with a metabolic map for visualization and a task list for model validation.
(ZIP)

Figure S1 Proteome comparison of genomes in *Fungi*. ALR (the ratio of alignment length to query sequence length): >0.50, identity: >0.40. The red shades refer to protein homology that can found within a genome (paralog). The green shades refer to protein homology that can found between two genomes (ortholog).
(PDF)

Figure S2 Agreement of model simulations with experimental fermentation data. Data from glucose-limited chemostat with defined medium containing glucose, inorganic salts and phenoxyacetate.
(PDF)

Table S1 Reactions which were excluded from the general KEGG model after running *removeBadRxns*. 72 reactions were unbalanced, general or polymer reactions and were therefore correctly removed. 7 reactions were correct in KEGG, but were removed because they lacked metabolite composition (it is a setting in *removeBadRxns* whether it is allowed to remove such reactions).
(PDF)

Table S2 Comparison of an automatically reconstructed model for *S. cerevisiae* to a published model of the same organism (iIN800) in terms of included genes. The table shows the genes that are unique to either the automatically reconstructed or the manually reconstructed model, and a classification of the genes into groups that reflect how well suited they are for being included in a GEM. Genes labeled as “enzymatic” should be included, while all other groups should probably be excluded. For iIN800 some enzymatic genes are further classified as “polymer”, “lipid” or “membrane”. These are parts of metabolism where an automatically generated model from KEGG would have particular drawbacks compared to a manually reconstructed model. “Polymer” corresponds mainly to genes involved in sugar polymer metabolism, which is an area that contains many unbalanced reactions in KEGG. Such reactions were excluded in the validation, so the corresponding genes could not be included. The same is true for “lipid”, where the reactions contain many general metabolites, which also results in excluded reactions. “Membrane” corresponds to reactions which depend on any one metabolite in different compartments. This compartmentalization information is absent in KEGG so such a reaction would read, for example, A+B = >A+C. “A” here might mean “A(cytosolic)” and “A(mitochondrial)”, but since that information is missing, the equation becomes incorrect and it is therefore excluded. “Signaling” corresponds to proteins which are primarily involved in signaling, even though they might have an enzymatic capability.
(PDF)

Table S3 Metabolites which could be synthesized in the automatically reconstructed *S. cerevisiae* model from minimal media (glucose, phosphate, sulfate, NH3, oxygen, 4-aminobenzoate, riboflavin, thiamine, biotin, folate, and nicotinate). Uptake of the carriers carnitine and acyl-carrier protein was allowed for modeling purposes (many compounds are bound to them and therefore net synthesis of these compounds is not possible without them).
(PDF)

Table S4 New metabolites which could be synthesized in the automatically reconstructed *S. cerevisiae* model from minimal media after gap-filling. These metabolites were all present in the model before the addition of new reactions.
(PDF)

Table S5 Reactions which were added to the automatically reconstructed *S. cerevisiae* model by *fillGaps* **.** Out of the 45 added reactions 17 has evidence to support that they should be included in the model, 9 has inconclusive of missing evidence, and 19 should not have been included in the model.
(PDF)

Table S6 Genes where their corresponding reactions were localized to the mitochondria after running *predictLocalization* (transport cost = 0.1). The color indicates whether the gene product is mitochondrial in SGD, where green means that it does, yellow that it is unclear, and red that it does not.
(PDF)

Table S7 Reactions which cannot carry flux even when all uptake reactions are unconstrained.
(PDF)

Table S8 Comparison of metabolic models.
(PDF)

Table S9 Biomass composition calculations for *P. chrysogenum*.
(PDF)

Table S10 Reactions with significantly higher flux in DS17690 compared to Wis 54-1255 where the corresponding genes are also up-regulated. Ranked by significance (p<0.05).
(PDF)

Table S11 Reporter metabolites when comparing the DS17690 and Wis 54-1255 strains. Ranked by significance. Top 40 best scoring metabolites are shown.
(PDF)
