# Supplementary material for: The RAVEN Toolbox and Its Use for Generating a Genome-scale Metabolic Model for Penicillium chrysogenum
Source: PLoS Comput Biol. 2013 Mar 21;9(3):e1002980. doi: 10.1371/journal.pcbi.1002980 (PMC3605104; doi:10.1371/journal.pcbi.1002980)
Supplement: Figure S1 — Proteome comparison of genomes in Fungi. ALR (the ratio of alignment length to query sequence length): >0.50, identity: >0.40. The red shades refer to protein homology that can found within a genome (paralog). The green shades refer to protein homology that can found between two genomes (ortholog). (PDF) [file pcbi.1002980.s002.pdf]

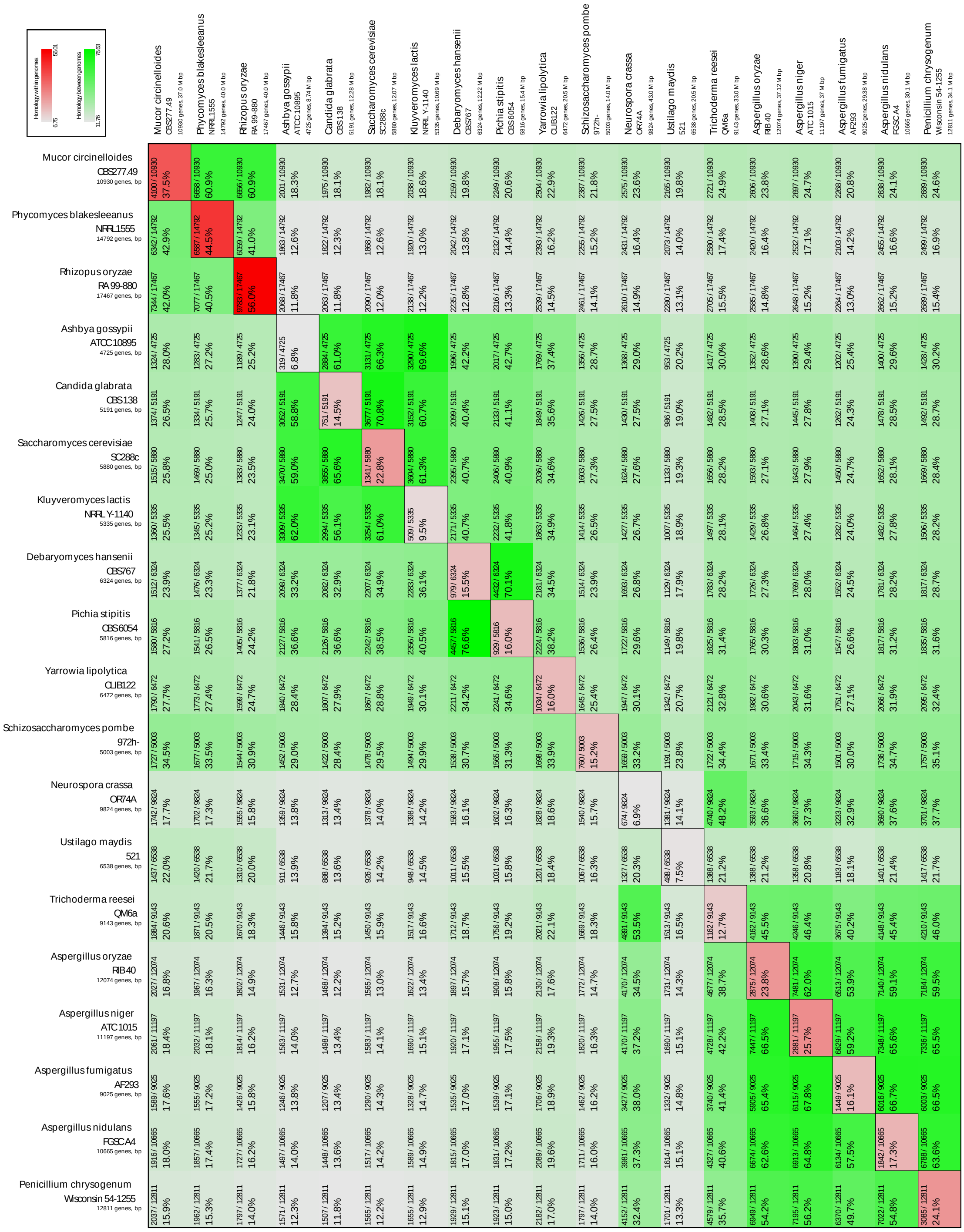

Figure S1. Proteome comparison of genomes in *Fungi*. ALR (the ratio of alignment length to query sequence length): >0.50, identity: >0.40. The red shades refer to protein homology that can found within a genome (paralog). The green shades refer to protein homology that can found between two genomes (ortholog).
