# Supplementary material for: The RAVEN Toolbox and Its Use for Generating a Genome-scale Metabolic Model for Penicillium chrysogenum
Source: PLoS Comput Biol. 2013 Mar 21;9(3):e1002980. doi: 10.1371/journal.pcbi.1002980 (PMC3605104; doi:10.1371/journal.pcbi.1002980)
Supplement: Figure S2 — Agreement of model simulations with experimental fermentation data. Data from glucose-limited chemostat with defined medium containing glucose, inorganic salts and phenoxyacetate. (PDF) [file pcbi.1002980.s003.pdf]

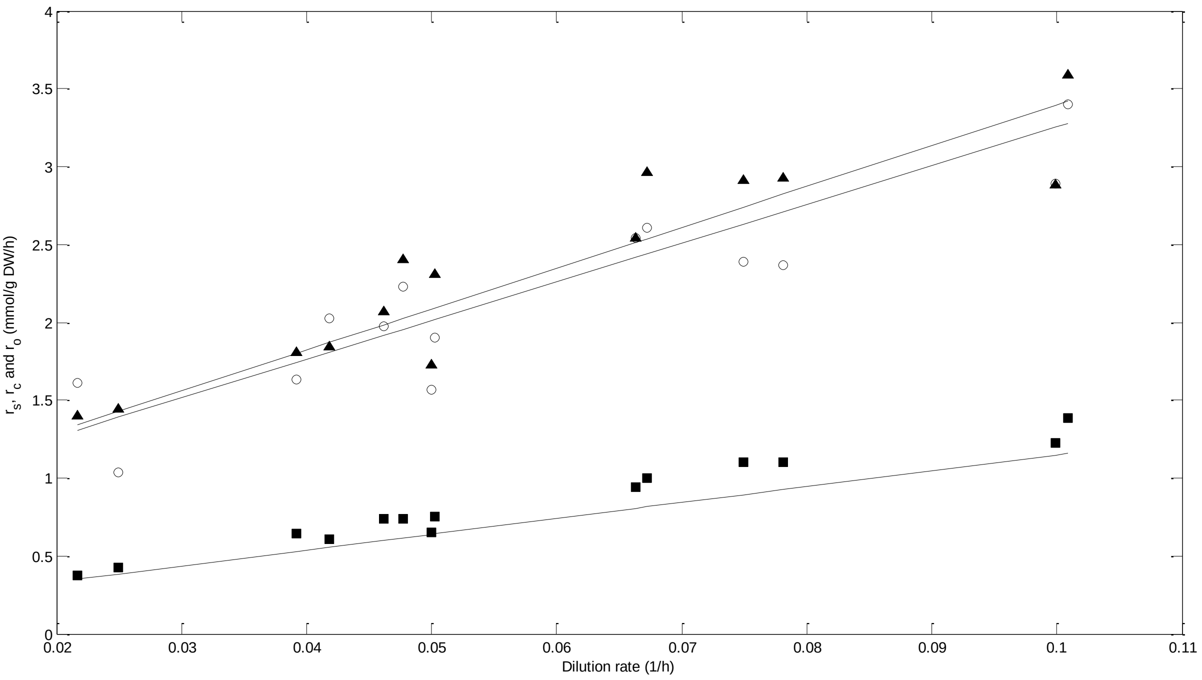

Figure S2. Agreement of model simulations with experimental fermentation data. Data from glucose-limited chemostat with defined medium containing glucose, inorganic salts and phenoxyacetate [1].

References

1. Nielsen JH (1995) Physiological engineering aspects of penicillium chrysogenum. Denmark: Polyteknisk forlag. x, 223 p. p.
