# Supplementary material for: The RAVEN Toolbox and Its Use for Generating a Genome-scale Metabolic Model for Penicillium chrysogenum
Source: PLoS Comput Biol. 2013 Mar 21;9(3):e1002980. doi: 10.1371/journal.pcbi.1002980 (PMC3605104; doi:10.1371/journal.pcbi.1002980)
Supplement: Table S1 — Reactions which were excluded from the general KEGG model after running removeBadRxns. 72 reactions were unbalanced, general or polymer reactions and were therefore correctly removed. 7 reactions were correct in KEGG, but were removed because they lacked metabolite composition (it is a setting in removeBadRxns whether it is allowed to remove such reactions). (PDF) [file pcbi.1002980.s004.pdf]

Table S1. Reactions which were excluded from the general KEGG model after running *removeBadRxns*. 72 reactions were unbalanced, general or polymer reactions and were therefore correctly removed. 7 reactions were correct in KEGG, but were removed because they lacked metabolite composition (it is a setting in *removeBadRxns* whether it is allowed to remove such reactions).

Reaction ID	Reaction status
R00019	Correct reaction with missing information
R00296	General
R00305	General
R00459	Unbalanced
R00476	General
R00785	Correct reaction with missing information
R00798	General
R00861	General
R01120	General
R01303	General
R01350	General
R02110	Polymer
R02550	Unbalanced
R02663	General
R02818	Unbalanced
R03141	Unbalanced
R03172	General
R03532	General
R03533	General
R03536	General
R03569	Unbalanced
R04098	General
R04644	General
R05325	Unbalanced
R05472	Unbalanced
R05476	Unbalanced
R05539	Unbalanced
R05666	Unbalanced
R05745	General
R05875	Correct reaction with missing information
R06061	Polymer
R06084	Polymer
R06103	Polymer
R06113	Polymer
R06159	Polymer
R06185	Polymer
R06186	Polymer
R06333	Unbalanced
R06334	Unbalanced
R06445	Unbalanced
R06565	Unbalanced
R06579	Unbalanced
R06580	Unbalanced
R06584	Unbalanced
R06612	Polymer
R06635	Unbalanced
R06722	Polymer
R06727	Unbalanced
R06745	Unbalanced
R06955	Unbalanced
R06959	Unbalanced
R07157	Correct reaction with missing information
R07436	Unbalanced
R07482	Unbalanced

R07738	Unbalanced
R07810	Polymer
R07943	Unbalanced
R07945	Unbalanced
R08011	Unbalanced
R08122	Unbalanced
R08147	Unbalanced
R08456	Unbalanced
R08539	Correct reaction with missing information
R08567	Correct reaction with missing information
R08586	Unbalanced
R08812	Unbalanced
R09066	Unbalanced
R09071	Correct reaction with missing information
R09089	Unbalanced
R09093	Unbalanced
R09142	Unbalanced
R09145	Unbalanced
R09146	Unbalanced
R09147	Unbalanced
R09149	Unbalanced
R09151	Unbalanced
R09210	Unbalanced
R09218	Unbalanced
R09317	General
