# Supplementary material for: The RAVEN Toolbox and Its Use for Generating a Genome-scale Metabolic Model for Penicillium chrysogenum
Source: PLoS Comput Biol. 2013 Mar 21;9(3):e1002980. doi: 10.1371/journal.pcbi.1002980 (PMC3605104; doi:10.1371/journal.pcbi.1002980)
Supplement: Table S2 — Comparison of an automatically reconstructed model for S. cerevisiae to a published model of the same organism (iIN800) in terms of included genes. The table shows the genes that are unique to either the automatically reconstructed or the manually reconstructed model, and a classification of the genes into groups that reflect how well suited they are for being included in a GEM. Genes labeled as “enzymatic” should be included, while all other groups should probably be excluded. For iIN800 some enzymatic genes are further classified as “polymer”, “lipid” or “membrane”. These are parts of metabolism where an automatically generated model from KEGG would have particular drawbacks compared to a manually reconstructed model. “Polymer” corresponds mainly to genes involved in sugar polymer metabolism, which is an area that contains many unbalanced reactions in KEGG. Such reactions were excluded in the validation, so the corresponding genes could not be included. The same is true for “lipid”, where the reactions contain many general metabolites, which also results in excluded reactions. “Membrane” corresponds to reactions which depend on any one metabolite in different compartments. This compartmentalization information is absent in KEGG so such a reaction would read, for example, A+B = >A+C. “A” here might mean “A(cytosolic)” and “A(mitochondrial)”, but since that information is missing, the equation becomes incorrect and it is therefore excluded. “Signaling” corresponds to proteins which are primarily involved in signaling, even though they might have an enzymatic capability. (PDF) [file pcbi.1002980.s005.pdf]

Table S2. Comparison of an automatically reconstructed model for *S. cerevisiae* to a published model of the same organism (iIN800) in terms of included genes [1]. The table shows the genes that are unique to either the automatically reconstructed or the manually reconstructed model, and a classification of the genes into groups that reflect how well suited they are for being included in a GEM. Genes labeled as "enzymatic" should be included, while all other groups should probably be excluded. For iIN800 some enzymatic genes are further classified as "polymer", "lipid" or "membrane". These are parts of metabolism where an automatically generated model from KEGG would have particular drawbacks compared to a manually reconstructed model. "Polymer" corresponds mainly to genes involved in sugar polymer metabolism, which is an area that contains many unbalanced reactions in KEGG. Such reactions were excluded in the validation, so the corresponding genes could not be included. The same is true for "lipid", where the reactions contain many general metabolites, which also results in excluded reactions. "Membrane" corresponds to reactions which depend on any one metabolite in different compartments. This compartmentalization information is absent in KEGG so such a reaction would read, for example, A + B => A + C. "A" here might mean "A(cytosolic)" and "A(mitochondrial)", but since that information is missing, the equation becomes incorrect and it is therefore excluded. "Signaling" corresponds to proteins which are primarily involved in signaling, even though they might have an enzymatic capability.

**Only in automatically reconstructed**	**Protein type**	**Only in iIN800**	**Protein type**
YAL039C	enzymatic	Q0045	enzymatic (membrane)
YAL060W	enzymatic	YAR073W	nonfunctional
YAL061W	enzymatic	YAR075W	nonfunctional
YBL064C	enzymatic	YBL099W	enzymatic (membrane)
YBL080C	enzymatic	YBR011C	enzymatic
YBL082C	enzymatic	YBR023C	enzymatic (polymer)
YBR004C	enzymatic	YBR034C	signaling
YBR070C	enzymatic	YBR036C	enzymatic (polymer)
YBR092C	enzymatic	YBR038W	enzymatic (polymer)
YBR093C	enzymatic	YBR184W	nonfunctional
YBR110W	enzymatic	YBR199W	enzymatic (polymer)
YBR111C	enzymatic	YBR284W	nonfunctional
YBR213W	enzymatic	YCR024C-A	nonenzymatic
YBR221C	enzymatic	YDL040C	signaling
YBR229C	enzymatic	YDL085W	enzymatic (membrane)
YBR243C	enzymatic	YDL090C	signaling
YBR245C	nonenzymatic	YDL100C	signaling
YCL009C	enzymatic	YDR007W	possible strain issue
YCL017C	enzymatic	YDR072C	enzymatic (polymer)
YCL052C	enzymatic	YDR261C	enzymatic (polymer)
YCR105W	enzymatic	YDR284C	enzymatic (lipid)
YCR107W	enzymatic	YDR315C	enzymatic
YDL036C	enzymatic	YDR399W	enzymatic
YDL080C	enzymatic	YDR503C	enzymatic (lipid)
YDL141W	enzymatic	YEL017C-A	nonenzymatic
YDL236W	enzymatic	YEL024W	enzymatic (membrane)
YDL238C	enzymatic	YEL047C	enzymatic
YDL243C	enzymatic	YER003C	enzymatic
YDL246C	enzymatic	YER024W	enzymatic (membrane)
YDR009W	signaling	YFL045C	nonenzymatic
YDR036C	enzymatic	YFR015C	enzymatic (polymer)
YDR148C	enzymatic	YFR055W	enzymatic
YDR173C	enzymatic	YGL008C	nonenzymatic

YDR178W	enzymatic	YGL248W	signaling
YDR196C	enzymatic	YGR032W	enzymatic (polymer)
YDR248C	unknown	YGR147C	signaling
YDR302W	enzymatic	YGR282C	enzymatic (polymer)
YDR305C	enzymatic	YHR042W	enzymatic
YDR368W	enzymatic	YHR067W	enzymatic (lipid)
YDR380W	enzymatic	YHR068W	enzymatic
YDR402C	enzymatic	YIL073C	nonenzymatic
YDR428C	enzymatic	YIL167W	nonfunctional
YDR437W	enzymatic	YJL070C	putative enzyme
YDR440W	signaling	YJL126W	enzymatic
YDR453C	enzymatic	YJR051W	enzymatic
YDR465C	signaling	YJR066W	signaling
YDR487C	enzymatic	YJR073C	enzymatic (lipid)
YDR516C	unknown	YJR133W	enzymatic
YDR539W	enzymatic	YKL004W	enzymatic (lipid)
YEL029C	kinase	YKL055C	enzymatic (lipid)
YEL038W	enzymatic	YKL132C	putative enzyme
YEL042W	enzymatic	YKL192C	nonenzymatic
YEL070W	unknown	YKL203C	signaling
YER001W	enzymatic	YKL212W	signaling
YER005W	enzymatic	YLL012W	enzymatic (lipid)
YER141W	enzymatic	YLR020C	enzymatic (lipid)
YER168C	enzymatic	YLR172C	signaling
YER183C	enzymatic	YLR195C	signaling
YFL060C	unknown	YLR258W	enzymatic (polymer)
YFL061W	unknown	YLR284C	enzymatic (lipid)
YFR044C	enzymatic	YLR300W	enzymatic (polymer)
YGL022W	nonenzymatic	YLR342W	enzymatic (polymer)
YGL027C	enzymatic	YML120C	enzymatic (membrane)
YGL038C	enzymatic	YMR006C	enzymatic (lipid)
YGL047W	enzymatic	YMR008C	enzymatic (lipid)
YGL065C	enzymatic	YMR145C	enzymatic (membrane)
YGL142C	enzymatic	YMR267W	enzymatic
YGL154C	enzymatic	YMR272C	enzymatic (lipid)
YGL184C	enzymatic	YMR306W	enzymatic (polymer)
YGL257C	enzymatic	YMR316W	nonfunctional
YGR010W	enzymatic	YNL192W	enzymatic (polymer)
YGR036C	enzymatic	YNL202W	enzymatic (lipid)
YGR110W	enzymatic	YNL292W	signaling
YGR180C	enzymatic	YNL325C	signaling
YGR216C	enzymatic	YOL011W	enzymatic (lipid)
YGR227W	enzymatic	YOL065C	enzymatic (lipid)
YGR277C	enzymatic	YOL136C	enzymatic
YHR011W	enzymatic	YOR180C	maybe enzyme
YHR033W	unknown	YOR190W	nonenzymatic
YHR039C	unknown	YOR221C	enzymatic (lipid)
YHR047C	enzymatic	YPL036W	nonenzymatic
YHR104W	enzymatic	YPL053C	enzymatic (polymer)
YHR119W	signaling	YPL057C	enzymatic (lipid)
YHR179W	enzymatic	YPL110C	enzymatic (lipid)
YHR201C	enzymatic	YPL212C	signaling

YHR215W	enzymatic	YPL266W	signaling
YIL014W	enzymatic	YPL275W	not in S288C strain
YIL043C	enzymatic	YPL276W	not in S288C strain
YIL049W	enzymatic	YPR021C	nonenzymatic
YIL083C	enzymatic	YPR026W	enzymatic
YIL099W	enzymatic	YPR160W	enzymatic (polymer)
YIL164C	possible strain issue	YPR176C	signaling
YJL026W	enzymatic		
YJL045W	enzymatic		
YJL046W	signaling		
YJL060W	enzymatic		
YJL062W	nonenzymatic		
YJL071W	enzymatic		
YJL091C	enzymatic		
YJL097W	enzymatic		
YJL132W	unknown		
YJL168C	signaling		
YJR013W	enzymatic		
YJR024C	enzymatic		
YJR069C	enzymatic		
YJR096W	enzymatic		
YJR130C	enzymatic		
YJR131W	enzymatic		
YJR153W	enzymatic		
YKL087C	enzymatic		
YKL088W	enzymatic		
YKL148C	enzymatic		
YKL150W	enzymatic		
YKL157W	enzymatic		
YKL165C	nonenzymatic		
YKL215C	enzymatic		
YKL218C	enzymatic		
YKR043C	enzymatic		
YKR066C	enzymatic		
YKR072C	enzymatic		
YLL031C	enzymatic		
YLL041C	enzymatic		
YLL057C	enzymatic		
YLL058W	unknown		
YLR017W	enzymatic		
YLR070C	enzymatic		
YLR118C	signaling		
YLR164W	unknown		
YLR239C	signaling		
YLR345W	enzymatic		
YLR351C	nonenzymatic		
YLR410W	enzymatic		
YML028W	enzymatic		
YML086C	enzymatic		
YML087C	unknown		
YML125C	signaling		
YMR009W	enzymatic		

YMR020W	enzymatic
YMR095C	unknown
YMR099C	enzymatic
YMR118C	unknown
YMR226C	enzymatic
YMR278W	enzymatic
YMR281W	enzymatic
YMR318C	enzymatic
YMR323W	unknown
YNL036W	enzymatic
YNL038W	enzymatic
YNL045W	enzymatic
YNL048W	enzymatic
YNL168C	unknown
YNL219C	enzymatic
YNL274C	enzymatic
YNL331C	enzymatic
YNL334C	putative enzyme
YNL335W	unknown
YNR027W	enzymatic
YNR030W	enzymatic
YNR073C	enzymatic
YOL066C	enzymatic
YOL143C	enzymatic
YOL157C	enzymatic
YOR002W	enzymatic
YOR067C	enzymatic
YOR108W	enzymatic
YOR120W	enzymatic
YOR125C	enzymatic
YOR136W	enzymatic
YOR149C	enzymatic
YOR175C	enzymatic
YOR196C	enzymatic
YOR251C	nonenzymatic
YOR269W	nonenzymatic
YOR274W	enzymatic
YOR303W	enzymatic
YOR304W	nonenzymatic
YOR326W	nonenzymatic
YPL017C	nonenzymatic
YPL069C	enzymatic
YPL076W	enzymatic
YPL082C	nonenzymatic
YPL096C-A	nonenzymatic
YPL171C	enzymatic
YPL175W	enzymatic
YPL206C	enzymatic
YPL227C	enzymatic
YPR002W	enzymatic
YPR073C	nonenzymatic
YPR118W	enzymatic

YPR127W	unknown
YPR140W	enzymatic
YPR184W	enzymatic

References

1. Nookaew I, Jewett MC, Meechai A, Thammarongtham C, Laoteng K, et al. (2008) The genome-scale metabolic model iIN800 of Saccharomyces cerevisiae and its validation: a scaffold to query lipid metabolism. BMC Syst Biol 2: 71.
