# Supplementary material for: The RAVEN Toolbox and Its Use for Generating a Genome-scale Metabolic Model for Penicillium chrysogenum
Source: PLoS Comput Biol. 2013 Mar 21;9(3):e1002980. doi: 10.1371/journal.pcbi.1002980 (PMC3605104; doi:10.1371/journal.pcbi.1002980)
Supplement: Table S3 — Metabolites which could be synthesized in the automatically reconstructed S. cerevisiae model from minimal media (glucose, phosphate, sulfate, NH3, oxygen, 4-aminobenzoate, riboflavin, thiamine, biotin, folate, and nicotinate). Uptake of the carriers carnitine and acyl-carrier protein was allowed for modeling purposes (many compounds are bound to them and therefore net synthesis of these compounds is not possible without them). (PDF) [file pcbi.1002980.s006.pdf]

Table S3. Metabolites which could be synthesized in the automatically reconstructed *S. cerevisiae* model from minimal media (glucose, phosphate, sulfate, NH3, oxygen, 4-aminobenzoate, riboflavin, thiamine, biotin, folate, and nicotinate [1]). Uptake of the carriers carnitine and acyl-carrier protein was allowed for modeling purposes (many compounds are bound to them and therefore net synthesis of these compounds is not possible without them).

**KEGG metabolite name**
(-)-Ureidoglycolate
(2E)-Octadecenoyl-CoA
(2R,3S)-3-Isopropylmalate
(2S)-2-Isopropyl-3-oxosuccinate
(3R)-3-Hydroxybutanoyl-[acyl-carrier protein]
(3R)-3-Hydroxydecanoyl-[acyl-carrier protein]
(3R)-3-Hydroxyoctanoyl-[acyl-carrier protein]
(3R)-3-Hydroxypalmitoyl-[acyl-carrier protein]
(3R)-3-Hydroxytetradecanoyl-[acyl-carrier protein]
(9Z)-Octadecenoic acid
(R)-2,3-Dihydroxy-3-methylbutanoate
(R)-2,3-Dihydroxy-3-methylpentanoate
(R)-2-Hydroxybutane-1,2,4-tricarboxylate
(R)-3-Hydroxy-3-methyl-2-oxopentanoate
(R)-3-Hydroxydodecanoyl-[acp]
(R)-3-Hydroxyhexanoyl-[acp]
(R)-4'-Phosphopantothenoyl-L-cysteine
(R)-5-Diphosphomevalonate
(R)-5-Phosphomevalonate
(R)-Mevalonate
(R)-Pantoate
(S)(+)-Allantoin
(S)-1-Pyrroline-5-carboxylate
(S)-2,3-Epoxysqualene
(S)-2-Aceto-2-hydroxybutanoate
(S)-2-Acetolactate
(S)-3-Hydroxy-3-methylglutaryl-CoA
(S)-3-Hydroxyhexadecanoyl-CoA
(S)-3-Methyl-2-oxopentanoic acid
(S)-Dihydroorotate
(S)-Malate
(Z)-But-1-ene-1,2,4-tricarboxylate
1-(2-Carboxyphenylamino)-1-deoxy-D-ribulose 5-phosphate
1-(5-Phospho-D-ribosyl)-5-amino-4-imidazolecarboxylate
1-(5-Phospho-D-ribosyl)-ATP
1-(5'-Phosphoribosyl)-5-amino-4-(N-succinocarboxamide)-imidazole
1-(5'-Phosphoribosyl)-5-amino-4-imidazolecarboxamide
1-(5'-Phosphoribosyl)-5-formamido-4-imidazolecarboxamide
10-Formyltetrahydrofolate
14-Demethyllanosterol
1D-myo-Inositol 1,3,4,5,6-pentakisphosphate
1D-myo-Inositol 1,4,5,6-tetrakisphosphate
1D-myo-Inositol 1,4-bisphosphate
1D-myo-Inositol 3-phosphate
2-(alpha-Hydroxyethyl)thiamine diphosphate
2-(Formamido)-N1-(5'-phosphoribosyl)acetamidine
2,3-Bisphospho-D-glycerate
2,3-Dihydroxy-3-methylbutanoate
2,5-Diamino-6-(5-phospho-D-ribitylamino)pyrimidin-4(3H)-one
2,5-Diamino-6-(5-phospho-D-ribosylamino)pyrimidin-4(3H)-one
2,5-Diamino-6-(5'-triphosphoryl-3',4'-trihydroxy-2'-oxopentyl)-amino-4-oxopyrimidine
2,5-Diaminopyrimidine nucleoside triphosphate
2,5-Dioxopentanoate
2-Acetolactate

2-Amino-3-carboxymuconate semialdehyde
2-Amino-4-hydroxy-6-(D-erythro-1,2,3-trihydroxypropyl)-7,8-dihydropteridine
2-Amino-4-hydroxy-6-(erythro-1,2,3-trihydroxypropyl)dihydropteridine triphosphate
2-Amino-4-hydroxy-6-hydroxymethyl-7,8-dihydropteridine
2-Amino-7,8-dihydro-4-hydroxy-6-(diphosphooxymethyl)pteridine
2-Dehydro-3-deoxy-D-arabino-heptonate 7-phosphate
2-Dehydropantoate
2-Isopropylmaleate
2-Keto-D-gluconic acid
2-Oxoadipate
2-Oxobutanoate
2-Oxoglutarate
2-Phenylacetamide
2-Phospho-D-glycerate
2-Phosphoglycolate
3-(4-Hydroxyphenyl)pyruvate
3-(Imidazol-4-yl)-2-oxopropyl phosphate
3',5'-Cyclic AMP
3',5'-Cyclic GMP
3-Carboxy-1-hydroxypropyl-ThPP
3-Dehydroquinate
3-Dehydroshikimate
3-Dehydrosphinganine
3-Fumarylpyruvate
3-Hydroxy-3-methyl-2-oxobutanoic acid
3-Hydroxyanthranilate
3-Hydroxy-L-kynurenine
3-Hydroxyoctadecanoyl-[acp]
3-Hydroxyoctadecanoyl-CoA
3-Keto-4-methylzymosterol
3-Methyl-2-oxobutanoic acid
3-Oxoadipyl-CoA
3-Oxodecanoyl-[acp]
3-Oxododecanoyl-[acp]
3-Oxohexadecanoyl-[acp]
3-Oxohexanoyl-[acp]
3-Oxooctanoyl-[acp]
3-Oxopropanoate
3-Oxostearoyl-[acp]
3-Oxostearoyl-CoA
3-Oxotetradecanoyl-[acp]
3'-Phosphoadenylyl sulfate
3-Phospho-D-glycerate
3-Phospho-D-glyceroyl phosphate
3-Phosphonooxypyruvate
4-(2-Amino-3-hydroxyphenyl)-2,4-dioxobutanoate
4-(2-Aminophenyl)-2,4-dioxobutanoate
4,4-Dimethyl-5alpha-cholesta-8,14,24-trien-3beta-ol
4alpha-Methylzymosterol
4alpha-Methylzymosterol-4-carboxylate
4-Amino-4-deoxychorismate
4-Aminobenzoate
4-Aminobutanoate
4-Phospho-L-aspartate
5-(5-Phospho-D-ribosylaminoformimino)-1-(5-phosphoribosyl)-imidazole-4-carboxamide
5,10-Methenyltetrahydrofolate
5,10-Methylenetetrahydrofolate
5-Amino-4-imidazolecarboxyamide
5-Amino-6-(1-D-ribitylamino)uracil
5-Amino-6-(5'-phospho-D-ribitylamino)uracil

5-Aminolevulinate
5-Formyltetrahydrofolate
5-Methyltetrahydrofolate
5-O-(1-Carboxyvinyl)-3-phosphoshikimate
5-Phospho-alpha-D-ribose 1-diphosphate
5-Phosphoribosylamine
5'-Phosphoribosylglycinamide
5'-Phosphoribosyl-N-formylglycinamide
6,7-Dimethyl-8-(D-ribityl)lumazine
6-Phospho-D-gluconate
Acetaldehyde
Acetate
Acetoacetyl-[acp]
Acetoacetyl-CoA
Acetyl adenylate
Acetyl-[acyl-carrier protein]
Acetyl-CoA
Acyl-carrier protein
Adenine
Adenosine
Adenosine 3',5'-bisphosphate
Adenylyl sulfate
ADP
Allantoate
all-trans-Decaprenyl diphosphate
all-trans-Hexaprenyl diphosphate
alpha,alpha-Trehalose
alpha,alpha'-Trehalose 6-phosphate
alpha-D-Galactose 1-phosphate
alpha-D-Glucose
alpha-D-Glucose 1,6-bisphosphate
alpha-D-Glucose 6-phosphate
alpha-D-Ribose 1-phosphate
alpha-Isopropylmalate
Aminoimidazole ribotide
AMP
Amylose
Anthranilate
ATP
beta-Alanine
beta-D-Fructose
beta-D-Fructose 1,6-bisphosphate
beta-D-Fructose 2,6-bisphosphate
beta-D-Fructose 6-phosphate
beta-D-Glucose
beta-D-Glucose 6-phosphate
Biotin
Biotinyl-5'-AMP
But-2-enoyl-[acyl-carrier protein]
Butyryl-[acp]
Carbamoyl phosphate
Carbonic acid
Carnitine
Carnosine
CDP
CDP-ethanolamine
Chorismate
Cinnavalininate
cis-Aconitate
Citrate

CMP
CO_2
CoA
Coproporphyrinogen III
CTP
Cyanamide
Cycloartenol
Cys-Gly
Cystathionine
Cytidine
Cytosine
D-4'-Phosphopantothenate
dADP
dAMP
dATP
dCDP
dCMP
dCTP
Deamino-NAD+
Decanoyl-[acp]
Dephospho-CoA
D-erythro-1-(Imidazol-4-yl)glycerol 3-phosphate
D-Erythrose 4-phosphate
D-Fructose
D-Fructose 1,6-bisphosphate
D-Fructose 1-phosphate
D-Fructose 6-phosphate
D-Galactose
dGDP
D-Gluconic acid
D-Glucono-1,5-lactone 6-phosphate
D-Glucosamine
D-Glucosamine 6-phosphate
D-Glucose
D-Glucose 1-phosphate
D-Glucose 6-phosphate
D-Glyceraldehyde
D-Glyceraldehyde 3-phosphate
D-Glycerate
dGMP
dGTP
Dihydrofolate
Dihydropteroate
Dimethylallyl diphosphate
Diphosphate
DL-Glutamate
D-myo-Inositol 1,4,5-trisphosphate
Dodecanoyl-[acyl-carrier protein]
D-Ribose
D-Ribose 5-phosphate
D-Ribulose 5-phosphate
D-Serine
D-Sorbitol
dTDP
dTMP
dTTP
dUDP
dUMP
dUTP
D-Xylose

D-Xylulose
D-Xylulose 5-phosphate
Ethanol
Ethanolamine
Ethanolamine phosphate
FAD
FADH2
FMN
Folate
Formamidopyrimidine nucleoside triphosphate
Formate
Formylanthranilate
Fumarate
Galactitol
gamma-L-Glutamyl-L-cysteine
GDP
Geranyl diphosphate
Geranylgeranyl diphosphate
Glutaminyl-tRNA
Glutaryl-CoA
Glutathione
Glutathione disulfide
Glycerol
Glycerone
Glycerone phosphate
Glycine
Glycolaldehyde
Glycolate
Glycyl-tRNA
Glyoxylate
GMP
GTP
Guanine
Guanosine
H+
H2O
HCO3-
Hexadecanal
Hexadecanoic acid
Hexadecanoyl-[acp]
Hexanoyl-[acp]
Homoisocitrate
Hydrogen peroxide
Hydrogen sulfide
Hydroxymethylbilane
Hydroxypyruvate
Hypoxanthine
IMP
Indole
Indoleglycerol phosphate
Inosine
Isocitrate
Isopentenyl diphosphate
L-2-Aminoadipate
L-2-Aminoadipate 6-semialdehyde
L-2-Aminoadipate adenylate
L-3,4-Dihydroxybutan-2-one 4-phosphate
L-Alanine
L-Alanyl-tRNA
L-Allothreonine

Lanosterol
L-Arginine
L-Arginyl-tRNA
L-Asparagine
L-Asparaginyl-tRNA
L-Aspartate
L-Aspartate 4-semialdehyde
L-Aspartyl-tRNA
L-Citrulline
L-Cystathionine
L-Cysteine
L-Cysteinyl-tRNA
L-Formylkynurenine
L-Glutamate
L-Glutamate 5-semialdehyde
L-Glutamine
L-Glutamyl 5-phosphate
L-Glutamyl-tRNA
L-Histidinal
L-Histidine
L-Histidinol
L-Histidinol phosphate
L-Histidyl-tRNA
L-Homocysteine
L-Homoserine
L-Isoleucine
L-Isoleucyl-tRNA
L-Kynurenine
L-Lysine
L-Lysyl-tRNA
L-Ornithine
L-Phenylalanine
L-Phenylalanyl-tRNA
L-Proline
L-Prolyl-tRNA
L-Selenocystathionine
L-Selenocysteine
L-Serine
L-Seryl-tRNA
L-Threonine
L-Threonyl-tRNA
L-Tryptophan
L-Tryptophanyl-tRNA
L-Tyrosine
L-Tyrosyl-tRNA
L-Valine
L-Valyl-tRNA
Malonyl-[acyl-carrier protein]
Malonyl-CoA
Maltose
Mannitol
Mercaptopyruvate
myo-Inositol
myo-Inositol 4-phosphate
N-(5'-Phospho-D-1'-ribulosylformimino)-5-amino-1-(5''-phospho-D-ribosyl)-4-imidazolecarboxamide
N-(5-Phospho-D-ribosyl)anthranilate
N-(L-Arginino)succinate
N6-(1,2-Dicarboxyethyl)-AMP
N6-(L-1,3-Dicarboxypropyl)-L-lysine
N-Acetyl-alpha-D-glucosamine 1-phosphate

N-Acetyl-D-glucosamine 6-phosphate
N-Acetyl-L-glutamate
N-Acetyl-L-glutamate 5-phosphate
N-Acetyl-L-glutamate 5-semialdehyde
N-Acetylornithine
NAD+
NADH
NADP+
NADPH
N-Carbamoyl-L-aspartate
NH3
Nicotinamide
Nicotinamide D-ribonucleotide
Nicotinate
Nicotinate D-ribonucleoside
Nicotinate D-ribonucleotide
N-Ribosylnicotinamide
O2.-
O-Acetylcarnitine
O-Acetyl-L-homoserine
O-Acetyl-L-serine
Octadecanoic acid
Octanoyl-[acp]
Oleoyl-CoA
O-Phospho-L-homoserine
O-Phospho-L-serine
Orotate
Orotidine 5'-phosphate
Orthophosphate
O-Succinyl-L-homoserine
Oxaloacetate
Oxaloglutarate
Oxalosuccinate
Oxygen
P1,P4-Bis(5'-adenosyl) tetraphosphate
Palmitoyl-CoA
Pantetheine 4'-phosphate
Pantothenate
Phenylacetaldehyde
Phenylacetic acid
Phenylpyruvate
Phosphoenolpyruvate
Phosphoribosyl-AMP
Phytosphingosine
Porphobilinogen
Prephenate
Presqualene diphosphate
Protoporphyrin
Protoporphyrinogen IX
Pseudouridine 5'-phosphate
Putrescine
Pyridoxal
Pyridoxal phosphate
Pyridoxamine
Pyridoxamine phosphate
Pyridoxine
Pyridoxine phosphate
Pyruvate
Quinolinate
Riboflavin

S-(Hydroxymethyl)glutathione
S-Adenosyl-L-homocysteine
Se-Adenosyl-L-selenohomocysteine
Sedoheptulose 1,7-bisphosphate
Sedoheptulose 7-phosphate
Selenide
Selenite
Selenohomocysteine
S-Formylglutathione
Shikimate
Shikimate 3-phosphate
sn-glycero-3-Phosphoethanolamine
sn-Glycerol 3-phosphate
Sphinganine
Sphinganine 1-phosphate
Squalene
S-Sulfo-L-cysteine
Starch
Stearoyl-CoA
Succinate
Succinate semialdehyde
Succinyl-CoA
Sucrose
Sucrose 6-phosphate
Sulfate
Sulfite
Tetradecanoyl-[acp]
Tetrahydrofolate
Tetrahydrofolyl-[Glu](2)
Thiamin diphosphate
Thiamin triphosphate
Thiamine
Thiosulfate
trans,trans-Farnesyl diphosphate
trans-Dec-2-enoyl-[acp]
trans-Dodec-2-enoyl-[acp]
trans-Hex-2-enoyl-[acp]
trans-Hexadec-2-enoyl-[acp]
trans-Hexadec-2-enoyl-CoA
trans-Oct-2-enoyl-[acp]
trans-Tetradec-2-enoyl-[acp]
Trehalose 6-phosphate
tRNA
tRNA containing 6-isopentenyladenosine
UDP
UDP-D-galactose
UDP-glucose
UDP-N-acetyl-D-glucosamine
UMP
Uracil
Urea
Urea-1-carboxylate
Uridine
Uroporphyrinogen III
UTP
Xanthine
Xanthosine
Xanthosine 5'-phosphate
Xylitol

References

1. McDonald PN (2001) Two-hybrid systems. Methods and protocols. Introduction. Methods Mol Biol 177: v-viii.
