# Supplementary material for: The RAVEN Toolbox and Its Use for Generating a Genome-scale Metabolic Model for Penicillium chrysogenum
Source: PLoS Comput Biol. 2013 Mar 21;9(3):e1002980. doi: 10.1371/journal.pcbi.1002980 (PMC3605104; doi:10.1371/journal.pcbi.1002980)
Supplement: Table S4 — New metabolites which could be synthesized in the automatically reconstructed S. cerevisiae model from minimal media after gap-filling. These metabolites were all present in the model before the addition of new reactions. (PDF) [file pcbi.1002980.s007.pdf]

Table S4. New metabolites which could be synthesized in the automatically reconstructed *S. cerevisiae* model from minimal media after gap-filling. These metabolites were all present in the model before the addition of new reactions.

**KEGG metabolite name**
(R)-Lactate
(R)-S-Lactoylglutathione
(S)-Lactate
(S)-Methylmalonyl-CoA
(Z)-But-2-ene-1,2,3-tricarboxylate
1,2-Dihydroxy-5-(methylthio)pent-1-en-3-one
2,3-Diketo-5-methylthiopentyl-1-phosphate
24-Methylenecycloartanol
2-Deoxy-D-ribose 1-phosphate
2-Deoxy-scyllo-inosamine
2-Deoxy-scyllo-inosose
2-Methylacetoacetyl-CoA
2-Methylcitrate
2-Oxoglutaramate
3-(Methylthio)propionic acid
3-Aminopropanal
3-Cyano-L-alanine
3-Sulfino-L-alanine
3-Sulfinylpyruvate
3-Sulfopyruvate
4-Aminobutyraldehyde
4-Coumarate
4-Hydroxystyrene
4-Methyl-2-oxopentanoate
4-Methylthio-2-oxobutanoic acid
5-Hydroxyconiferyl alcohol
5-Hydroxy-guaiacyl lignin
5'-Methylthioadenosine
ADP-ribose
alpha-Aminopropiononitrile
Aminoacetaldehyde
beta-D-Fructose 2-phosphate
Betaine
Betaine aldehyde
Caffeic aldehyde
Caffeoyl-CoA
CDP-choline
Choline
Choline phosphate
CO
Coniferyl alcohol
Coniferyl aldehyde
Cyanide
Deoxyadenosine
Deoxycytidine
Deoxyguanosine
Deoxyinosine
Deoxyuridine
Ferulate
gamma-Amino-gamma-cyanobutanoate
gamma-Glutamyl-beta-cyanoalanine
Guaiacyl lignin
Hydrogen cyanide
Hypotaurine
Inositol 1-phosphate
Lactaldehyde

L-Arogenate
L-Cysteate
L-Leucine
L-Leucyl-tRNA
L-Methionine
L-Methionyl-tRNA
L-Selenomethionine
Methaneselenol
Methylglyoxal
Methylselenopyruvate
N-Formylmethionyl-tRNA
p-Coumaroyl-CoA
Precorrin 2
Propanal
Propane-1,2-diol
Propanoate
Propanoyl-CoA
Propionyladenylate
S-Adenosyl-L-methionine
S-Adenosylmethioninamine
Se-Adenosylselenomethionine
Selenomethionyl-tRNA
Se-Methyl-L-selenocysteine
Sinapate
Sinapoyl aldehyde
Sinapyl alcohol
Sirohydrochlorin
S-Methyl-5-thio-D-ribose 1-phosphate
S-Methyl-5-thio-D-ribulose 1-phosphate
sn-glycero-3-Phosphocholine
Spermidine
Spermine
Syringyl lignin
Taurine
Thiocyanate
