# Supplementary material for: The RAVEN Toolbox and Its Use for Generating a Genome-scale Metabolic Model for Penicillium chrysogenum
Source: PLoS Comput Biol. 2013 Mar 21;9(3):e1002980. doi: 10.1371/journal.pcbi.1002980 (PMC3605104; doi:10.1371/journal.pcbi.1002980)
Supplement: Table S5 — Reactions which were added to the automatically reconstructed S. cerevisiae model by fillGaps . Out of the 45 added reactions 17 has evidence to support that they should be included in the model, 9 has inconclusive of missing evidence, and 19 should not have been included in the model. (PDF) [file pcbi.1002980.s008.pdf]

Table S5. Reactions which were added to the automatically reconstructed *S. cerevisiae* model by *fillGaps*. Out of the 45 added reactions 17 has evidence to support that they should be included in the model, 9 has inconclusive of missing evidence, and 19 should not have been included in the model.

KEGG reaction	EC number	Correctly added	Exists in iIN800	Comments
R00931	2.3.3.5	Yes	Yes, but only as a citrate synthase	YPR001W has methylcitrate synthase ability in addition to citrate synthase according to SGD
R02054	3.1.1.32	Yes	No	YOR022C has been suggested to have phospholipase A1 ability according to SGD
R01016	4.2.3.3	Yes	No	Methylglyoxal synthase exists in *S. cerevisiae [1]*
R02744	3.1.1.5	Yes	Yes	Lysophospholipase exist according to SGD. YMR008C, YOL011W and YMR006C were previously in iIN800 but not in automatically reconstructed model
R01664	3.1.3.5	Yes	Yes, but without associated genes	5'-nucleotidases exist according to BRENDA. YOR155C is a possible gene, but might be specific to IMP
R02730	3.1.3.54	Yes	No	Fructose-2,6-bisphosphate 6-phosphatase does not have an associated KO in KEGG, but the reference points to *S. cerevisiae*
R01650	1.5.3.11	Yes	Yes, but without associated genes	YMR020W is the *S. cerevisiae* polyamine oxidase
R01598	6.2.1.3	Yes	Yes	The reaction could be carried out by long-chain fatty acid-coa ligase which is already in the model
R00946	2.1.1.13	Yes	Yes, but without associated genes	YER091C is a cobalamin independent version (2.1.1.14)
R04861	-	Yes	No	Spontaneous
R08361	-	Yes	No	Spontaneous
R04931	2.1.1.9	Yes	No	YMR209C is a putative S-

				adenosylmethionine-dependent methyltransferase that matches according to SGD
R01320	2.1.1.71	Yes	No, but the responsible gene is present (but for the wrong EC number)	YJR073C is a hosphatidyl-N-methylethanolamine N-methyltransferase according to SGD
R03424	2.1.1.71	Yes	No, but the responsible gene is present (but for the wrong EC number)	YJR073C is a hosphatidyl-N-methylethanolamine N-methyltransferase according to SGD
R01731	2.6.1.57	Yes	Yes, with unclear EC numbers but the correct gene (YGL202W)	The gene also exists in the automatically reconstructed model, but was mapped to some other amino acid transferases
R09097	-	Yes	Yes	Propionaldehyde dehydrogenase function exists according to SGD. The general aldehyde dehydrogenases YMR170C, YMR169C, YOR374W, YER073W, YPL061W exists in the model but not with this reaction
R00544	-	Yes	Yes	Propionaldehyde dehydrogenase function exists according to SGD. The general aldehyde dehydrogenases YMR170C, YMR169C, YOR374W, YER073W, YPL061W exists in the model but not with this reaction
R00102	3.2.2.5	Uncertain	Yes, but without associated genes	Could not find any other evidence
R04443	-	Uncertain		no KO/EC
R07383	-	Uncertain		no KO/EC
R03714	-	Uncertain		no KO/EC
R01410	-	Uncertain		unknown reaction
R08890	2.6.1.-	Uncertain	Yes, but with uncomplete EC number	Unclear whether the reaction could be carried out by the same enzyme which is used

				in iIN800
R02434	-	Uncertain		no KO/EC
R08714	2.6.1.-	Uncertain	Yes, but with uncomplete EC number	Unclear whether the reaction could be carried out by the same enzyme which is used in iIN800
R03185	-	Uncertain		No EC number in KEGG, but YKR076W and YGR154C are associated to the GO term "glutathione dehydrogenase (ascorbate) activity" that fits the reaction
R04330	1.1.53	No	No	
R00095	1.6.5.4	No	No	
R08617	4.2.3.-	No	No	
R01267	4.2.1.65	No	No	
R07409	1.14.15.7	No	No	
R01182	2.7.1.64	No	No	
R00737	4.3.1.23	No	No	
R02593	1.1.1.195	No	No	
R03918	1.1.1.195	No	No	
R06573	1.14.13.-	No	No	
R06574	2.1.1.68	No	No	
R06577	2.1.1.68	No	No	
R01616	6.2.1.12	No	No	
R03524	4.4.1.9	No	No	
R02376	4.2.1.28	No	No	
R01941	1.2.1.44	No	No	
R01945	2.3.1.99	No	No	
R04342	2.3.1.133	No	No	
R07432	2.3.1.133	No	No	

References

1. Murata K, Fukuda Y, Watanabe K, Saikusa T, Shimosaka M, et al. (1985) Characterization of methylglyoxal synthase in Saccharomyces cerevisiae. Biochem Biophys Res Commun 131: 190-198.
