# Supplementary material for: The RAVEN Toolbox and Its Use for Generating a Genome-scale Metabolic Model for Penicillium chrysogenum
Source: PLoS Comput Biol. 2013 Mar 21;9(3):e1002980. doi: 10.1371/journal.pcbi.1002980 (PMC3605104; doi:10.1371/journal.pcbi.1002980)
Supplement: Table S6 — Genes where their corresponding reactions were localized to the mitochondria after running predictLocalization (transport cost = 0.1). The color indicates whether the gene product is mitochondrial in SGD, where green means that it does, yellow that it is unclear, and red that it does not. (PDF) [file pcbi.1002980.s009.pdf]

**Table S6.** Genes where their corresponding reactions were localized to the mitochondria after running *predictLocalization* (transport cost=0.1). The color indicates whether the gene product is mitochondrial in SGD, where green means that it does, yellow that it is unclear, and red that it does not.

| In common | Comments | Only in automatically reconstructed | Comments | Only in iIN800 | Comments |
|---|---|---|---|---|---|
| YAR035W | | YBL098W | | YBR029C | |
| YBL013W | | YBR003W | | YCR024C | |
| YBR026C | | YCL064C | | YDL182W | |
| YBR084W | | YDL168W | | YDR236C | |
| YBR263W | | YDR538W | | YER073W | |
| YCL004W | | YGR094W | | YGL125W | |
| YDL066W | | YHL032C | | YGR012W | |
| YDL142C | | YHR091C | | YJL200C | |
| YDL174C | | YHR190W | | YKL085W | |
| YDR019C | | YIL066C | | YLR304C | |
| YDR232W | | YKL067W | | YMR062C | |
| YDR234W | | YKL182W | | YMR207C | |
| YDR268W | | YLR100W | | YOR040W | |
| YER014W | | YLR305C | | YOR374W | |
| YER061C | | YML042W | | YPL188W | |
| YER069W | | YMR293C | | YEL041W | unknown |
| YER086W | | YOL059W | | YLR209C | intracellular |
| YER087W | | YOR241W | | YPL023C | unknown |
| YER170W | | YPL091W | | YER026C | |
| YER178W | | YPR006C | | YFL022C | |
| YFL018C | | YPR033C | | YFR047C | |
| YGR171C | | YPR113W | | YGL037C | |
| YGR244C | | YHR128W | intracellular | YHR074W | |
| YGR255C | | YKR069W | intracellular | YJR049C | |
| YHR037W | | YOL064C | unknown | YKL216W | |
| YHR106W | | YBR020W | | YLR060W | |
| YHR208W | | YBR205W | | YLR328W | |
| YIL094C | | YBR218C | | YOL097C | |
| YIL125W | | YBR252W | | YOR209C | |
| YIL155C | | YCR053W | | YOR236W | |
| YJR016C | | YDL021W | | YBR029C | |
| YKL029C | | YDL022W | | | |
| YKL106W | | YDR047W | | | |
| YKL141W | | YDR297W | | | |
| YKL194C | | YDR454C | | | |
| YLR089C | | YER070W | | | |
| YLR142W | | YFL001W | | | |
| YLR355C | | YGL001C | | | |
| YLR382C | | YGL012W | | | |
| YML054C | | YGR060W | | | |
| YML110C | | YGR087C | | | |
| YMR083W | | YGR175C | | | |
| YMR108W | | YHR063C | | | |
| YMR189W | | YIL145C | | | |
| YNL037C | | YJL068C | | | |
| YNL071W | | YJR078W | | | |
| YNL073W | | YKL001C | | | |
| YNL104C | | YKL008C | | | |
| YNL169C | | YKR009C | | | |
| YNR001C | | YLR027C | | | |
| YOL033W | | YLR058C | | | |
| YOL096C | | YLR303W | | | |
| YOL140W | | YMR015C | | | |

| | |
|---|---|
| YOR142W | YMR113W |
| YOR176W | YMR250W |
| YPL040C | |
| YPL097W | |
| YPL104W | |
| YPL262W | |
| YPR001W | |
| YPR047W | |
