# Supplementary material for: The RAVEN Toolbox and Its Use for Generating a Genome-scale Metabolic Model for Penicillium chrysogenum
Source: PLoS Comput Biol. 2013 Mar 21;9(3):e1002980. doi: 10.1371/journal.pcbi.1002980 (PMC3605104; doi:10.1371/journal.pcbi.1002980)
Supplement: Table S7 — Reactions which cannot carry flux even when all uptake reactions are unconstrained. (PDF) [file pcbi.1002980.s010.pdf]

Table S7. Reactions which cannot carry flux even when all uptake reactions are unconstrained.

Reaction ID	Description
r0071	1-aminocyclopropane-1-carboxylate aminohydrolase (isomerizing)
r0108	tartrate dehydrogenase
r0161	beta-glucuronidase
r0162	2-deoxy-D-gluconate:NAD+ 3-oxidoreductase
r0174	UDP-glucuronosyltransferase
r0213	poly(1,4-alpha-D-galacturonide) galacturonohydrolase
r0240	lactose galactohydrolase
r0356	ADP-ribose ribophosphohydrolase
r0405	guanosine 3'-diphosphate 5'-triphosphate 5'-phosphohydrolase
r0453	ATP:NADH 2'-phosphotransferase
r0454	NAD+ phosphohydrolase
r0514	L-cysteine:[ThiI] sulfurtransferase
r0515	ATP:4-amino-2-methyl-5-phosphomethylpyrimidine phosphotransferase
r0516	ATP:4-methyl-5-(2-hydroxyethyl)-thiazole 2-phosphotransferase
r0517	2-methyl-4-amino-5-hydroxymethylpyrimidine-diphosphate:4-methyl-5-(2-phosphoethyl)-thiazole 2-methyl-4-aminopyridine-5-methenyltransferase
r0534	myo-Inositol-hexakisphosphate 3-phosphohydrolase
r0573	agmatine amidinohydrolase
r0577	delta-1-pyrroline-5-carboxylate dehydrogenase
r0580	pyrroline-5-carboxylate reductase
r0581	trans-4-hydroxy-L-proline:NAD+ 5-oxidoreductase
r0597	5-methyltetrahydropteroyltri-L-glutamate:L-homocysteine S-methyltransferase
r0602	O-succinyl-L-homoserine succinate-lyase (adding cysteine)
r0603	O-succinyl-L-homoserine succinate-lyase (deaminating; 2-oxobutanoate-forming)
r0604	O-succinyl-L-homoserine succinate-lyase (adding hydrogen sulfide)
r0630	N,N-dimethylglycine oxidase
r0631	sarcosine:oxygen oxidoreductase (demethylating)
r0726	Indole-3-acetamide amidohydrolase
r0732	phenylethylalcohol:NAD+ oxidoreductase
r0752	3-indoleacetonitrile aminohydrolase
r0754	indole-3-acetamide hydro-lyase
