# Supplementary material for: The RAVEN Toolbox and Its Use for Generating a Genome-scale Metabolic Model for Penicillium chrysogenum
Source: PLoS Comput Biol. 2013 Mar 21;9(3):e1002980. doi: 10.1371/journal.pcbi.1002980 (PMC3605104; doi:10.1371/journal.pcbi.1002980)
Supplement: Table S8 — Comparison of metabolic models. (PDF) [file pcbi.1002980.s011.pdf]

Table S8. Comparison of metabolic models.

Organism	Model name	Metabolic genes	Metabolites[a]	Unique metabolic reactions[b]	References
A. nidulans	iHD666	666	543 (740)	789	[1]
A. niger	iMA871	871	782 (1047)	1194	[2]
A. oryzae	iWV1314	1314	808 (1102)	1243	[3]
P. chrysogenum	iAL1006	1006	849 (1235)	1471	This study

[a]Number of chemically distinct metabolites, not counting presence in multiple compartments. Metabolite number in parenthesis counting presence in multiple compartment

[b]Unique reactions are defined as reactions being biochemically unique in their own compartment or transport reactions. Isoenzymes are not included in this number. Exchange reactions are not included in this number.

References

1. David H, Ozcelik IS, Hofmann G, Nielsen J (2008) Analysis of Aspergillus nidulans metabolism at the genome-scale. BMC Genomics 9: 163.
2. Andersen MR, Nielsen ML, Nielsen J (2008) Metabolic model integration of the bibliome, genome, metabolome and reactome of Aspergillus niger. Mol Syst Biol 4: 178.
3. Vongsangnak W, Olsen P, Hansen K, Krogsgaard S, Nielsen J (2008) Improved annotation through genome-scale metabolic modeling of Aspergillus oryzae. BMC Genomics 9: 245.
