# Supplementary material for: The RAVEN Toolbox and Its Use for Generating a Genome-scale Metabolic Model for Penicillium chrysogenum
Source: PLoS Comput Biol. 2013 Mar 21;9(3):e1002980. doi: 10.1371/journal.pcbi.1002980 (PMC3605104; doi:10.1371/journal.pcbi.1002980)
Supplement: Table S9 — Biomass composition calculations for P. chrysogenum. (PDF) [file pcbi.1002980.s012.pdf]

Table S9. Biomass composition calculations for *P. chrysogenum.*

**[a]BIOMASS COMPOSITION (g/gDW)**	
Protein	0.45
RNA	0.08
DNA	0.01
Phospholipids	0.035
Sterolesters	0.01
Triacylglycerol[b]	0.005
Cell wall	0.22
Glycogen	0.03
Free amino acids	0.04
Free nucleotides	0.02
Metabolites	0.02

[a]If nothing else is stated the composition is taken from Table 4.13 in Physiological Engineering Aspects of Penicillin chrysogenum [1]
[b]This proportion is assumed to contain mono-, di-, and triacylglycerides as well as free fatty acids

AMINO ACIDS AND PROTEIN

	Molecular weight (g/mol)	Protein composition (mol/mol amino acids)[a]	Scaled protein composition (mol/mol amino acids)[d]	Protein composition (mmol/g protein)	Free amino acid pool (mol/mol free amino acids)[e,f]	Scaled free amino acid composition (mol/mol free amino acids)[d]	Amino acid pool composition (mmol/g free amino acids)
L-alanine	89.09	0.071	0.071753411	0.583943653	0.13	0.1363398	1.04484604
L-2-aminoadipate	161.15	0	0	0	0.006	0.00629261	0.04822366
L-arginine	174.2	0.0675	0.068216271	0.555157698	0.026	0.02726796	0.20896921
L-asparagine[b]	132.12	0.017125	0.017306721	0.140845564	0.034	0.0356581	0.27326743
L-aspartate[b]	133.11	0.051375	0.051920162	0.422536693	0.058	0.06082853	0.46616208
L-cysteine	121.16	0.018	0.018191006	0.148042053	0.005	0.00524384	0.04018639
L-glutamate[c]	147.13	0.088125	0.089060131	0.724789217	0.254	0.266387	2.04146841
L-glutamine[c]	146.15	0.029375	0.02968671	0.241596406	0.166	0.17409544	1.33418802
glycine	75.07	0.0755	0.076301162	0.620954166	0.032	0.03356057	0.25719287
L-histidine	155.16	0.025	0.025265285	0.205613962	0.028	0.0293655	0.22504376
L-isoleucine	131.18	0.042	0.04244568	0.345431457	0.013	0.01363398	0.1044846
L-leucine	131.18	0.0845	0.085396665	0.694975193	0.016	0.01678028	0.12859644
L-lysine	146.19	0.0755	0.076301162	0.620954166	0.022	0.02307289	0.1768201

L-methionine	149.21	0.054	0.054573017	0.444126159	0.005	0.00524384	0.04018639
L-phenylalanine	165.19	0.0335	0.033855483	0.27552271	0.005	0.00524384	0.04018639
L-proline	115.13	0.0515	0.052046488	0.423564762	0.0515	0.05401154	0.41391978
L-serine	105.09	0.0515	0.052046488	0.423564762	0.047	0.04929208	0.37775203
L-threonine	119.12	0.0535	0.054067711	0.440013879	0.031	0.0325118	0.24915559
L-tryptophan	204.23	0.007	0.00707428	0.057571909	0.002	0.00209754	0.01607455
L-tyrosine	118.19	0.0235	0.023749368	0.193277125	0.006	0.00629261	0.04822366
L-valine	117.15	0.07	0.070742799	0.575719094	0.016	0.01678028	0.12859644
**TOTAL**		0.9895	1		0.9535	1	

Molecular weight of protein (g/mol)	122.8772852
Molecular weight of free amino acid pool (g/mol)	130.4879339

[a]Data from Table 4.11 in Physiological Engineering Aspects of *Penicillin chrysogenum [1]*. Averaged composition from two studies (Jørgensen 1993;[2]
[b]The pool of aspartate and asparagine is measured. A ratio of 3:1 aspartate/asparagine is assumed (Oura, 1972)
[c]The pool of glutamate and glutamine is measured. A ratio of 3:1 glutamate/glutamine is assumed (Oura, 1972)
[d]Scaled composition to deal with the molecular balance not closing
[e]Data from Table 4.7 in Physiological Engineering Aspects of *Penicillin chrysogenum* [1]. Original data from Jørgensen (1993)
[f]Proline cannot be measured with the applied method. Assumes the same composition as in protein

DNA AND RNA

	RNA composition (mmol/g RNA)[a]		DNA composition (mmol/g DNA)[a]
AMP	0.79	dAMP	0.79
GMP	0.89	dGMP	0.86
CMP	0.61	dCMP	0.86
UMP	0.81	dTMP	0.79

[a]Following the reasoning in section 4.3.3 in Physiological Engineering Aspects of *Penicillin chrysogenum* [1]

FREE RIBONUCLEOTIDES

	Free ribonucleotide composition (mmol/g DW)[a]	Molecular weight (g/mol)	Composition (mmol/g free ribonucleotides)
GTP	0.0033	532.18	0.213396447
ATP	0.0225	507.18	1.454975775
UTP	0.00145	484.14	0.093765105
CTP	0.0033	483.16	0.213396447

[a]Following the reasoning in section 4.3.2 in Physiological Engineering Aspects of *Penicillin chrysogenum* [1]. Average values are used.

LIPIDS

	Molecular weight (g/mol)[a]	Fatty acid composition (mol/mol total fatty acids)[b]	Scaled fatty acid composition (mol/mol fatty acids)[c]	Free fatty acid composition (mmol/g free fatty acids)
pentadecanoyl	225.3925	0.0125	0.011927481	0.042968034
palmitoyl	239.4192	0.2425	0.23139313	0.833579865
hexadecenoyl	237.4032	0.03	0.028625954	0.103123282
stearoyl	267.4726	0.09	0.085877863	0.309369847
octadecenoyl	265.4566	0.047	0.044847328	0.161559809
octadecadienoyl	263.4406	0.48	0.458015267	1.649972517
arachidate	295.526	0.146	0.139312977	0.501866641
**TOTAL**		1.048	1	

**Average molecular weights (g/mol):**		Phospholipid composition (mol/mol total phospholipid)[d]	Phospholipid composition (mmol/g total phospholipid)	Acylglyceride composition (mol/mol acylglyceride)[d.f]	Acylglyceride composition (mmol/g total acylglyceride)	Ergosterol/ergosterol ester composition (mol/mol total ergosterol)[d]	Ergosterol/ergosterol ester composition (mmol/g total ergosterol)	Glucolipid composition (mol/gDW)[d.e]
acyl group	261.589634							
free fatty acids	277.589634			0.210607316	0.379742043			
monoglycerides	352.677734			0.351683043	0.634113001			
diglycerides	613.259368			0.039816898	0.07179309			
triglycerides	873.8410021			0.397892743	0.717432831			
phosphatidylserine	779.308468	0.006873576	0.00886945					
phosphatidylcholines	779.393868	0.293170461	0.378298093					
phosphatidylethanolamines	736.305768	0.666430527	0.859941335					
ergosterol	396.6516					0.77238095	1.69394811	
ergosterol ester	657.233234					0.22761905	0.49920296	
digalactosyl-diacylglycerol	675.958134							0.007859
trigalactosyldimannosylinositol-P-ceramide	2038.883868	9.57323E-05	0.00012353					
cerebrin 1	596.9949							0.00003
cerebrin 2	580.9949							0.000031
galactosylceramides	747.1284							0.000025
glucocerebroside 1	928.2966							0.000024

glucocerebroside 2	926.2806			0.000024
cardiolipin	1502.506836	0.033429704	0.043136656	

Molecular weight of phospholipid (g/mol)	774.972082
Molecular weight of acylglycerides (g/mol)	554.6062653
Molecular weight of sterol esters (g/mol)	455.9649434

[a]Composition calculated only for the acyl chain, not for the fatty acid (lacks the carboxyl group)
[b]Total fatty acid composition for growth on glucose as sole carbon source [3]
[c]Scaled composition to deal with the molecular balance not closing
[d]Assumes the same composition as in the A. niger model
[e]No reliable data could be found on the percentage of glucolipids in *Penicillium chrysogenum.* It is therefore assumed that the composition and amount is the same as in *Aspergillius niger* (see the A. niger model [4])
[f]The free fatty acid proportion differ between *Penicillium chrysogenum* and *Aspergillius niger* but it is assumed that the proportion relative to the acylglycerides is the same

**CELLWALL AND STORAGE[a]**	Molecular weight of monomer in cell wall (without UDP/GDP) (g/mol)[b]	Cell wall monomer composition (mol/mol total monomers)[b]	Cell wall monomer composition (mmol/g cell wall monomers)
UDP-N-acetyl-D-glucosamine	214.1596	0.27184466	1.54208087
UDP-glucose	162.1442	0.495145631	2.808790156
UDP-D-galactose	162.1442	0.145631068	0.826114752
GDP-mannose	162.1442	0.058252427	0.330445901
trehalose	162.1442	0.029126214	0.16522295

Molecular weight of cell wall and storage (mmol/g)	176.2843087

[a]The cell wall composition of *Penicillium chrysogenum* is not well researched and there is conflicting data. The cell wall composition if therefore expressed as a polymerization of the monomers.
[b]Following the reasoning in section 4.3.3 in Physiological Engineering Aspects of *Penicillin chrysogenum* [1]

POLYMERISATION COSTS[a]

DNA	11.22
RNA	7.44
Protein (mmol ATP/g protein)	34.9942627

[a]Following the reasoning in section 4.3.3 in Physiological Engineering Aspects of *Penicillin chrysogenum* [1]

References

1. Nielsen JH (1995) Physiological engineering aspects of penicillium chrysogenum. Denmark: Polyteknisk forlag. x, 223 p. p.
2. Jaklitsch WM, Hampel W, Rohr M, Kubicek CP, Gamerith G (1986) alpha-Aminoadipate pool concentration and penicillin biosynthesis in strains of Penicillium chrysogenum. Can J Microbiol 32: 473-480.
3. Divakaran P, Modak MJ (1968) Fatty acid composition of mycelium of Penicillium chrysogenum grown in different carbohydrates as a sole source of carbon. Experientia 24: 1102.
4. Andersen MR, Nielsen ML, Nielsen J (2008) Metabolic model integration of the bibliome, genome, metabolome and reactome of Aspergillus niger. Mol Syst Biol 4: 178.
