# Supplementary material for: The RAVEN Toolbox and Its Use for Generating a Genome-scale Metabolic Model for Penicillium chrysogenum
Source: PLoS Comput Biol. 2013 Mar 21;9(3):e1002980. doi: 10.1371/journal.pcbi.1002980 (PMC3605104; doi:10.1371/journal.pcbi.1002980)
Supplement: Table S10 — Reactions with significantly higher flux in DS17690 compared to Wis 54-1255 where the corresponding genes are also up-regulated. Ranked by significance (p<0.05). (PDF) [file pcbi.1002980.s013.pdf]

Table S10. Reactions with significantly higher flux in DS17690 compared to Wis 54-1255 where the corresponding genes are also up-regulated (as reported by the algorithm by [1]). Ranked by significance ($p<0.05$)

Reaction ID	Reaction name
r0601	O-acetyl-L-homoserine:hydrogen sulfide S-(3-amino-3-carboxypropyl)transferase
r0606	L-cystathionine cysteine-lyase (deaminating; 2-oxobutanoate-forming)
r0747	phenylacetate:CoA ligase
r1408	sulfate permease
r0648	L-valine:2-oxoglutarate aminotransferase
r0812	isopenicillin-N synthase
r0622	L-homoserine:NAD+ oxidoreductase
r1144	adenosine 3',5'-bisphosphate,sulfite:oxidized-thioredoxin oxidoreductase
r0619	3-phosphoserine:2-oxoglutarate aminotransferase
r0075	2-hydroxybutane-1,2,3-tricarboxylate hydro-lyase
r1150	adenosine 3',5'-bisphosphate 3'-phosphohydrolase
r0620	O-phospho-L-serine phosphohydrolase
r0101	acetyl-CoA:oxaloacetate C-acetyltransferase
r0074	propanoyl-CoA:oxaloacetate C-propanoyltransferase
r0814	L-2-aminohexanedioate:L-cysteine:L-valine ligase (AMP-forming, valine-inverting)
r0465	pyruvate:pyruvate acetaldehydetransferase (decarboxylating)
r0656	(R)-2,3-dihydroxy-3-methylbutanoate hydro-lyase
r0076	(2S,3R)-3-hydroxybutane-1,2,3-tricarboxylate pyruvate-lyase (succinate-forming)
r0618	3-phospho-D-glycerate:NAD+ 2-oxidoreductase
r1147	ATP:adenylylsulfate 3'-phosphotransferase
r0374	ATP:AMP phosphotransferase
r0127	ubiquinol:ferricytochrome-c oxidoreductase
r0546	L-aspartate-4-semialdehyde:NADP+ oxidoreductase
r1317	ammonium permease
r0128	H+-transporting two-sector ATPase
r1149	hydrogen-sulfide:NADP+ oxidoreductase
r0632	L-serine hydro-lyase (adding homocysteine; L-cystathionine-forming)
r0419	NADPH:oxidized-thioredoxin oxidoreductase
r1155	ADP:sulfate adenylyltransferase
r0547	ATP:L-aspartate 4-phosphotransferase
r1178	ADP/ATP/phosphate mitochondrial shuttle
r0600	acetyl-CoA:L-homoserine O-acetyltransferase
r0114	diphosphate phosphohydrolase
r0803	isopenicillin N amidohydrolase
r0815	acyl-CoA:6-APA acyltransferase
r0541	L-aspartate:2-oxoglutarate aminotransferase

References

1. Bordel S, Agren R, Nielsen J (2010) Sampling the solution space in genome-scale metabolic networks reveals transcriptional regulation in key enzymes. PLoS Comput Biol 6: e1000859.
