# Supplementary material for: The RAVEN Toolbox and Its Use for Generating a Genome-scale Metabolic Model for Penicillium chrysogenum
Source: PLoS Comput Biol. 2013 Mar 21;9(3):e1002980. doi: 10.1371/journal.pcbi.1002980 (PMC3605104; doi:10.1371/journal.pcbi.1002980)
Supplement: Table S11 — Reporter metabolites when comparing the DS17690 and Wis 54-1255 strains. Ranked by significance. Top 40 best scoring metabolites are shown. (PDF) [file pcbi.1002980.s014.pdf]

Table S11. Reporter metabolites when comparing the DS17690 and Wis 54-1255 strains [1]. Ranked by significance. Top 40 best scoring metabolites are shown

**Metabolite name**	**P-value**
N-[L-5-amino-5-carboxypentanoyl]-L-cysteinyl-D-valine[c]	0 .00292
sulfite[c]	0 .00387
chitosan[e]	0 .00795
glutathione[c]	0 .01265
aminoacetaldehyde[c]	0 .01458
D-glucosamine[e]	0 .01651
1D-myo-inositol 3-phosphate[c]	0 .01842
(S)-1-pyrroline-5-carboxylate[c]	0 .02040
L-1-pyrroline-3-hydroxy-5-carboxylate[c]	0 .02120
phenylacetate[p]	0 .02120
D-glucosamine 6-phosphate[c]	0 .02121
phenylacetyl-CoA[p]	0 .02122
ADP[p]	0 .02356
phosphate[p]	0 .02391
D-glucosamine[c]	0 .02391
taurine[c]	0 .02717
N-formimidoyl-L-glutamate[c]	0 .02757
maltotriose[c]	0 .02767
(S)-3-(5-oxo-4,5-dihydro-3H-imidazol-4-yl)propanoate[c]	0 .02767
adenosine 3',5'-bisphosphate[c]	0 .02913
L-valine[c]	0 .02956
succinate semialdehyde[c]	0 .03015
D-galactonate[c]	0 .03175
carbamate[c]	0 .03331
cyanate[c]	0 .03375
2-dehydro-3-deoxy-D-galactonate[c]	0 .03375
2-dehydro-3-deoxy-D-gluconate[c]	0 .03462
maltose[c]	0 .03462
phosphate[c]	0 .03498
5-oxo-L-proline[c]	0 .03580
L-asparagine[c]	0 .03656
L-glutaminyl-tRNA(Gln)[c]	0 .03718
gamma-L-glutamyl-L-cysteine[c]	0 .03885
L-cysteinylglycine[c]	0 .04005
R-S-alanylglycine[c]	0 .04478
phenylacetyl-CoA[c]	0 .04478
L-isoleucine[c]	0 .05046
L-isoleucyl-tRNA(Ile)[c]	0 .05523
L-tyrosine[c]	0 .05523
thioredoxin disulfide[c]	0 .05746
thioredoxin dithiol[c]	0 .05858

References

1. Patil KR, Nielsen J (2005) Uncovering transcriptional regulation of metabolism by using metabolic network topology. Proc Natl Acad Sci U S A 102: 2685-2689.
